# NBI-921352, a first-in-class, Na_V_1.6 selective, sodium channel inhibitor that prevents seizures in *Scn8a* gain-of-function mice, and wild-type mice and rats

**DOI:** 10.7554/eLife.72468

**Published:** 2022-03-02

**Authors:** JP Johnson, Thilo Focken, Kuldip Khakh, Parisa Karimi Tari, Celine Dube, Samuel J Goodchild, Jean-Christophe Andrez, Girish Bankar, David Bogucki, Kristen Burford, Elaine Chang, Sultan Chowdhury, Richard Dean, Gina de Boer, Shannon Decker, Christoph Dehnhardt, Mandy Feng, Wei Gong, Michael Grimwood, Abid Hasan, Angela Hussainkhel, Qi Jia, Stephanie Lee, Jenny Li, Sophia Lin, Andrea Lindgren, Verner Lofstrand, Janette Mezeyova, Rostam Namdari, Karen Nelkenbrecher, Noah Gregory Shuart, Luis Sojo, Shaoyi Sun, Matthew Taron, Matthew Waldbrook, Diana Weeratunge, Steven Wesolowski, Aaron Williams, Michael Wilson, Zhiwei Xie, Rhena Yoo, Clint Young, Alla Zenova, Wei Zhang, Alison J Cutts, Robin P Sherrington, Simon N Pimstone, Raymond Winquist, Charles J Cohen, James R Empfield

**Affiliations:** 1 In Vitro Biology, Xenon Pharmaceuticals Inc Burnaby Canada; 2 Chemistry, Xenon Pharmaceuticals Inc Burnaby Canada; 3 In Vivo Biology, Xenon Pharmaceuticals Inc Burnaby Canada; 4 Compound Properties, Xenon Pharmaceuticals Inc Burnaby BC Canada; 5 Translational Drug Development, Xenon Pharmaceuticals Inc Burnaby Canada; 6 Scientific Affairs, Xenon Pharmaceuticals, Inc Burnaby BC Canada; 7 Executive Team, Xenon Pharmaceuticals Inc Burnaby Canada; Washington University in St. Louis United States; The University of Texas at Austin United States

**Keywords:** epilepsy, SCN8A, NBI-921352, anti-seizure medicine, Mouse, Rat

## Abstract

NBI-921352 (formerly XEN901) is a novel sodium channel inhibitor designed to specifically target Na_V_1.6 channels. Such a molecule provides a precision-medicine approach to target *SCN8A*-related epilepsy syndromes (*SCN8A*-RES), where gain-of-function (GoF) mutations lead to excess Na_V_1.6 sodium current, or other indications where Na_V_1.6 mediated hyper-excitability contributes to disease (Gardella and Møller, 2019; Johannesen et al., 2019; Veeramah et al., 2012). NBI-921352 is a potent inhibitor of Na_V_1.6 (IC_50_0.051 µM), with exquisite selectivity over other sodium channel isoforms (selectivity ratios of 756 X for Na_V_1.1, 134 X for Na_V_1.2, 276 X for Na_V_1.7, and >583 Xfor Na_V_1.3, Na_V_1.4, and Na_V_1.5). NBI-921352is a state-dependent inhibitor, preferentially inhibiting inactivatedchannels. The state dependence leads to potent stabilization of inactivation, inhibiting Na_V_1.6 currents, including resurgent and persistent Na_V_1.6 currents, while sparing the closed/rested channels. The isoform-selective profile of NBI-921352 led to a robust inhibition of action-potential firing in glutamatergic excitatory pyramidal neurons, while sparing fast-spiking inhibitory interneurons, where Na_V_1.1 predominates. Oral administration of NBI-921352 prevented electrically induced seizures in a *Scn8a* GoF mouse,as well as in wild-type mouse and ratseizure models. NBI-921352 was effective in preventing seizures at lower brain and plasma concentrations than commonly prescribed sodium channel inhibitor anti-seizure medicines (ASMs) carbamazepine, phenytoin, and lacosamide. NBI-921352 waswell tolerated at higher multiples of the effective plasma and brain concentrations than those ASMs. NBI-921352 is entering phase II proof-of-concept trials for the treatment of *SCN8A-*developmental epileptic encephalopathy (*SCN8A*-DEE) and adult focal-onset seizures.

## Introduction

Na_V_1.6 voltage-gated sodium channels are widely expressed in the brain and are important contributors to neural excitability (Meisler, 2019; Royeck et al., 2008).Mutations in the *SCN8A* gene result in malfunction of Na_V_1.6 sodium channels and cause a spectrum of *SCN8A*-related syndromes in humans, and disruptions of mouse Na_V_1.6 likewise disrupt normal physiology (Burgess et al., 1995; Gardella and Møller, 2019; Johannesen et al., 2019; Meisler, 2019; Veeramah et al., 2012; Wagnon et al., 2015). Variants of Na_V_1.6 channels can result in either gain or loss of function. Loss-of-function (LoF) variants in humans are generally associated with autism spectrum disorders with cognitive and developmental delay without epilepsy (Inglis et al., 2020; Liu et al., 2019), but, in some cases, can lead to late-onset seizures.In mice, LoF variants of Na_V_1.6 lead to motor impairment but increase seizure resistance (Hawkins et al., 2011; Martin et al., 2007). Gain-of-function (GoF) variants in human *SCN8A* generally result in early-onset *SCN8A*-related epilepsy syndromes (*SCN8A*-RES). The most severe of these epilepsy syndromes is *SCN8A* developmental and epileptic encephalopathy (*SCN8A*-DEE) (Gardella and Møller, 2019; Hammer et al., 2016; Johannesen et al., 2019). Most *SCN8A*-RES patients carry de novo heterozygous missense variants that lead to a gain of function of the Na_V_1.6 channel, although inherited and bi-allelic variants have been reported (Gardella and Møller, 2019; Wengert et al., 2019).*SCN8A*-DEE patients present early in life with seizure onset usually occurring in the first year of life. After seizure onset, patients begin to miss developmental milestones and display additional symptoms, including cognitive and motor delay, hypotonia, and cortical blindness. *SCN8A*-DEE individuals are predisposed to early death, including sudden unexplained death in epilepsy (SUDEP). While *SCN8A*-RESpatients often have treatment-resistant seizures, many can achieve seizure reduction or seizure freedom upon treatment with anti-seizure medicines (ASMs) that non-selectively inhibit voltage-gated sodium channels, like phenytoin (Boerma et al., 2016; Braakman et al., 2017). *SCN8A*-RES patients may require doses that are higher than those prescribed for most epilepsy patients and, as a result, can be more prone to drug-related adverse events (Boerma et al., 2016; Gardella and Møller, 2019). Even with high doses and multiple ASMs, many patients continue to have uncontrolled seizures as well as extensive comorbidities. The aggressive pharmacotherapy required to protect *SCN8A*patients from life-threatening seizures often comes with attendant side effects that would not be tolerated in less severely impacted populations.

Existing sodium channel inhibitor ASMs are nonselective, blocking all voltage-gated sodium channel isoforms at similar plasma or brain concentrations. This lack of selectivity likely limits the benefits of sodium channel inhibitors since LoF variants of Na_V_1.1 are known toimpair inhibitory interneuron function and causegeneralized epilepsy with seizures plus (GEFS+) and *SCN1A*-DEE (Dravet Syndrome) (Catterall et al., 2010; Claes et al., 2001; Escayg et al., 2000; Gennaro et al., 2003).Thus, inhibiting Na_V_1.1 may counter the benefit of inhibiting the sodium channels of excitatory neurons. Inhibiting Na_V_1.4 and Na_V_1.5 currents is also undesirable since those channels are critical for facilitating contraction of skeletal and cardiac muscles, respectively (Chen et al., 1998; Ptácek et al., 1991; Rojas et al., 1991).

We hypothesized that a selective inhibitor of Na_V_1.6 could provide a safer and more effective treatment for patients with *SCN8A*-RES and might also be more broadly efficacious in more common forms of epilepsy. An extensive medicinal-chemistry effort produced NBI-921352, the firstpotent and selective inhibitor of Na_V_1.6 channels (Neurocrine, 2019). We explored the profile of NBI-921352 in vitro, ex vivo and in threepreclinical in vivo rodent seizure models, including electrically induced seizure assays in genetically engineered mice bearing heterozygous*Scn8a* GoFNa_V_1.6 channels (N1768D), as well as in wild-type mice and rats.

## Results

### In vitro Na_V_ potency and selectivity

**Figure 1.**
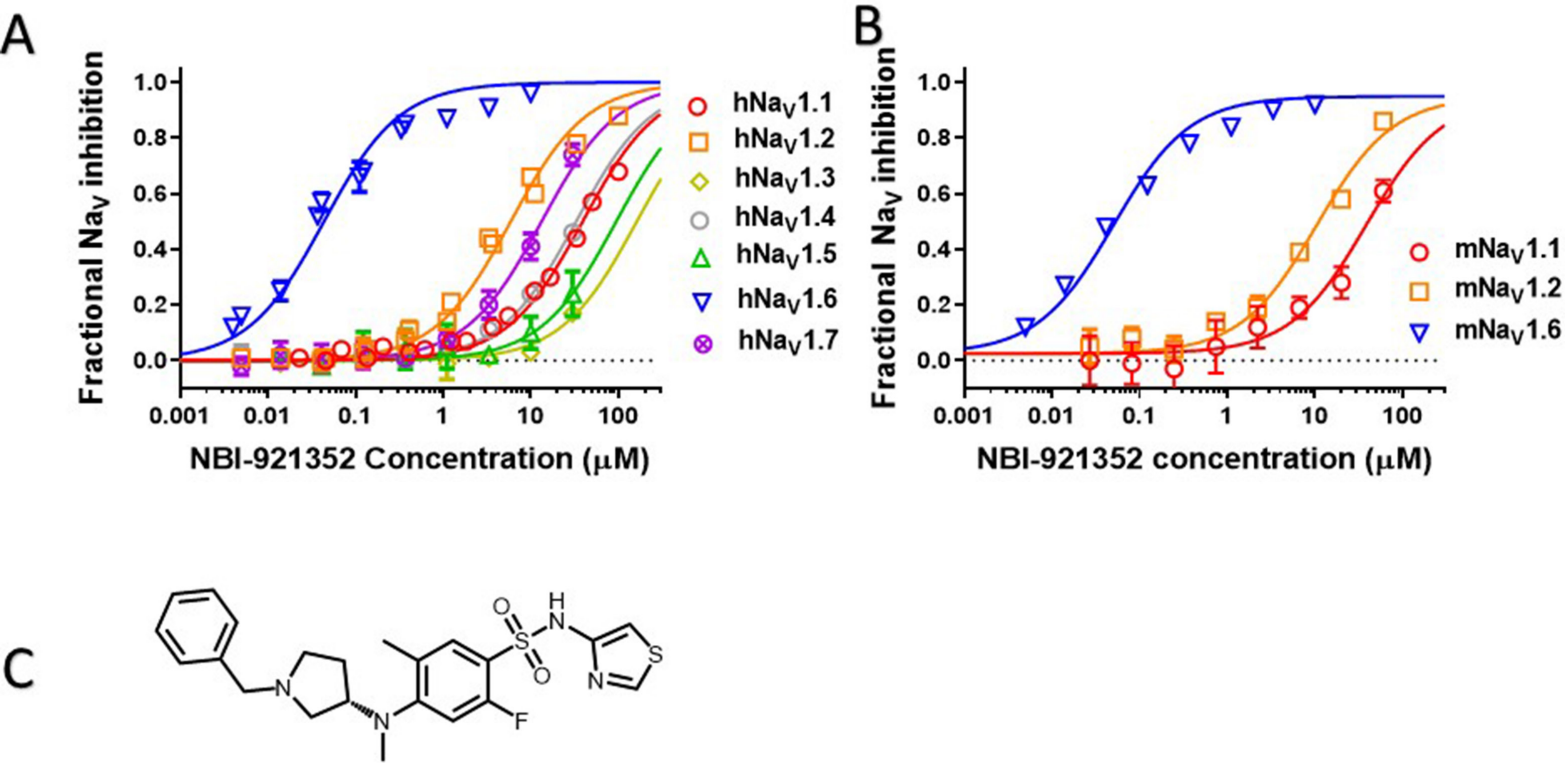
Potency and isoform selectivity of NBI-921352 for human and mouse Na_V_ channels. Concentration-response curves were generated by automated patch-clamp electrophysiology using the SophionQube. Concentration-response curves were generated for human (**A**) or mouse (**B**) Na_V_ channel isoforms heterologously expressed in HEK293 cells. The analysis included only those cells that met pre-specified acceptance criteria for seal quality, current amplitude, and series resistance. Normalized data from all cell recordings at a concentration were grouped together and plotted with GraphPad Prism 8. Details regarding the number of cells analyzed for each Na_V_ channel and concentration can be found in the source data sheet. Error bars indicating the standard error of the mean fraction were plotted for all points, but, in some cases, they were smaller than the data point symbols and, therefore, not visible. The chemical structure of NBI-921352 is shown (**C**). Figure 1—source data 1.Quantification of potency and isoform selectivity of NBI-921352.

Human Na_V_ channel isoforms hNa_V_1.1, hNa_V_1.2, hNa_V_1.3, hNa_V_1.4, hNa_V_1.5, hNa_V_1.6, and hNa_V_1.7 were heterologously expressed in HEK-293 cells, and the potency and isoformselectivity of NBI-921352 (Figure 1, Table 1) was determined by automated patch-clamp techniques.NBI-921352 potently inhibitedhNa_V_1.6 channel currents with an inhibitory concentration 50% (IC_50_)of 0.051 µM (95% CI: 0.030–0.073 µM; N = 3) calculated from three biological replicates. Inhibition of other human Na_V_1.X isoforms required higher concentrations of NBI-921352 with IC_50_’s of 39 µM (95% CI: 31–47 µM; N = 3)for hNa_V_1.1, 6.9 µM (95% CI: 1.6–12 µM; N = 3) for hNa_V_1.2, > 30 µM for hNa_V_1.3, > 30 µM for hNa_V_1.4, > 30 µM for hNa_V_1.5, and 14 µM (95% CI: 6.4–22 µM; N = 3) for hNa_V_1.7. These potencies provide selectivity ratios for hNa_V_1.6 versus the other hNa_V_ isoforms (IC_50_ hNa_V_1.X / IC_50_ hNa_V_1.6) of 756 (Na_V_1.1), 134 (Na_V_1.2), 276 (Na_V_1.7) and >583 (Na_V_1.3, Na_V_1.4, Na_V_1.5). Inhibition of Na_V_1.8 and Na_V_1.9 was not assessed since both channels arebelieved to be limited to peripheral sensory neurons and are not expected tohave much impact on the efficacy and tolerability of sodium channel inhibitors in epilepsy. Inhibition of Na_V_1.8 and Na_V_1.9 might reduce pain perception, but this would not be considered a significant liability for an anti-seizure medicine.

**Figure 1—figure supplement 1.**
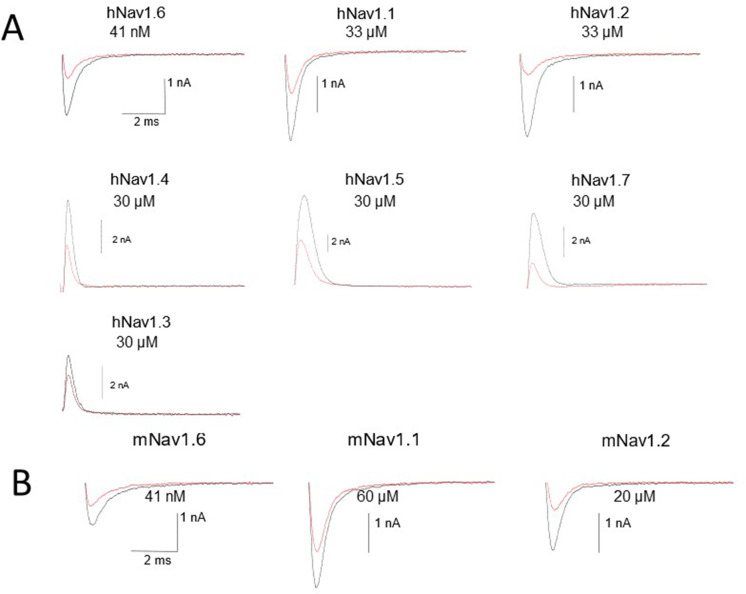
Representative raw traces of voltage-clamp recordings of control and the concentrations close to the NBI-921352 IC_50_ for all tested sodium channel isoforms in Figure 1. Note that in some cases currents are outward because the sodium gradients were reversed. This was done to improve assay robustness and reproducibility. See methods for details. We have found that current direction does not impact pharmacology for inhibitors, like NBI-921352, that bind the VSD4 site (not shown).

**Figure 1—figure supplement 2.**
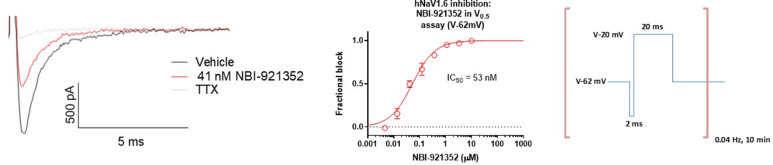
Potency of NBI-921352 measured form a holding potential approximating the V0.5 of inactivation for Na_V_1. 6. For the “V0.5” assay the membrane was held at –62 mV for Nav1.6 and the protocol shown above was applied. The solutions used were the same as for hNav1.6 in the standard assay. The protocol consisted of a 0.04 Hz repeating test pulse to –20 mV for 20ms to assess current inhibition preceded by a brief 2ms recovery pulse to –120 mV that was included to increase stability of the currents over time and boost the current size. The protocol was run in vehicle conditions at 0.1 Hz for 150 seconds compound to establish the baseline for each cell prior to the addition of compound for 10 min at a pulse frequency of 0.04 Hz. Full inhibition was defined by the addition of 300 nM TTX and data were processed in the same way as for the V-45 standard assay described in Figure 1. Figure 1—figure supplement 2—source data 1.Quantification of potency with alternate voltage protocol.

**Table 1.** Potency and isoform selectivity of NBI-921352 for human and mouse Na_V_ channels. Note that IC_50_s for the neuronal sodium channels, Na_V_ 1.1, Na_V_ 1.2, and Na_V_1.6, have been more accurately defined than those for non-neuronal sodium channels. Explicit IC_50_’s for Na_V_1.3, Na_V_1.4, and Na_V_1.5 were not determined since the inhibition at the highest concentration tested (30 µM) was <50%. IC_50_’s are the mean of 3 separate biological replicates of the IC_50_ determinations for each channel. The error is shown as the 95% confidence interval of the mean IC_50_. None of the other tested isoforms displayed IC_50_’s within the 95% confidence interval of the Na_V_1.6 IC_50_ and the confidence intervals were well separated as well.

	Na_V_1.6	Na_V_1.1	Na_V_1.2	Na_V_1.3	Na_V_1.4	Na_V_1.5	Na_V_1.7
**Human IC_50_ (µM**)	0.051	39	6.9	> 30	> 30	> 30	14
**95%** CI	0.030–0.073	31–47	1.6–12	-	-	-	6.4–22
**Human Selectivity hNa_V_1.X / hNa_V_1.6**	1	756	134	> 583	> 583	> 583	276
**Mouse IC_50_ (µM**)	0.058	41	11				
**Mouse Selectivity mNa_V_1.X / mNa_V_1.6**	1	709	191				

Since we intended to evaluate in vivo effects of NBI-921352 in mouse seizure models, we also assessed the potency of NBI-921352 in the mouse Na_V_ isoforms that are most highly expressed in the brain, Na_V_1.6, Na_V_1.1, and Na_V_1.2. The potency and selectivity in mouse Na_V_ channels closely paralleled that seen in the human orthologues with IC_50_’s of 0.058 µM (95% CI: 0.046–0.070 µM; N = 3) for mNa_V_1.6, 41 µM (95% CI: 30–52 µM; N = 3) for mNa_V_1.1, and 11 µM (95% CI: 8.2–14 µM; N = 3) for mNa_V_1.2. Selectivity ratios (IC_50_ mNa_V_1.X / IC_50_ mNa_V_1.6) were 709 (Na_V_1.1), and 191 (Na_V_1.2). These data indicate that NBI-921352 potently inhibits both human and mouse Na_V_1.6 channels, and that it does so at concentrations ≥ 134 fold lower than for any of the other channel isoforms tested.

### NBI-921352 inhibited patient-identified variants of Na_V_1.6 channels

**Figure 2.**
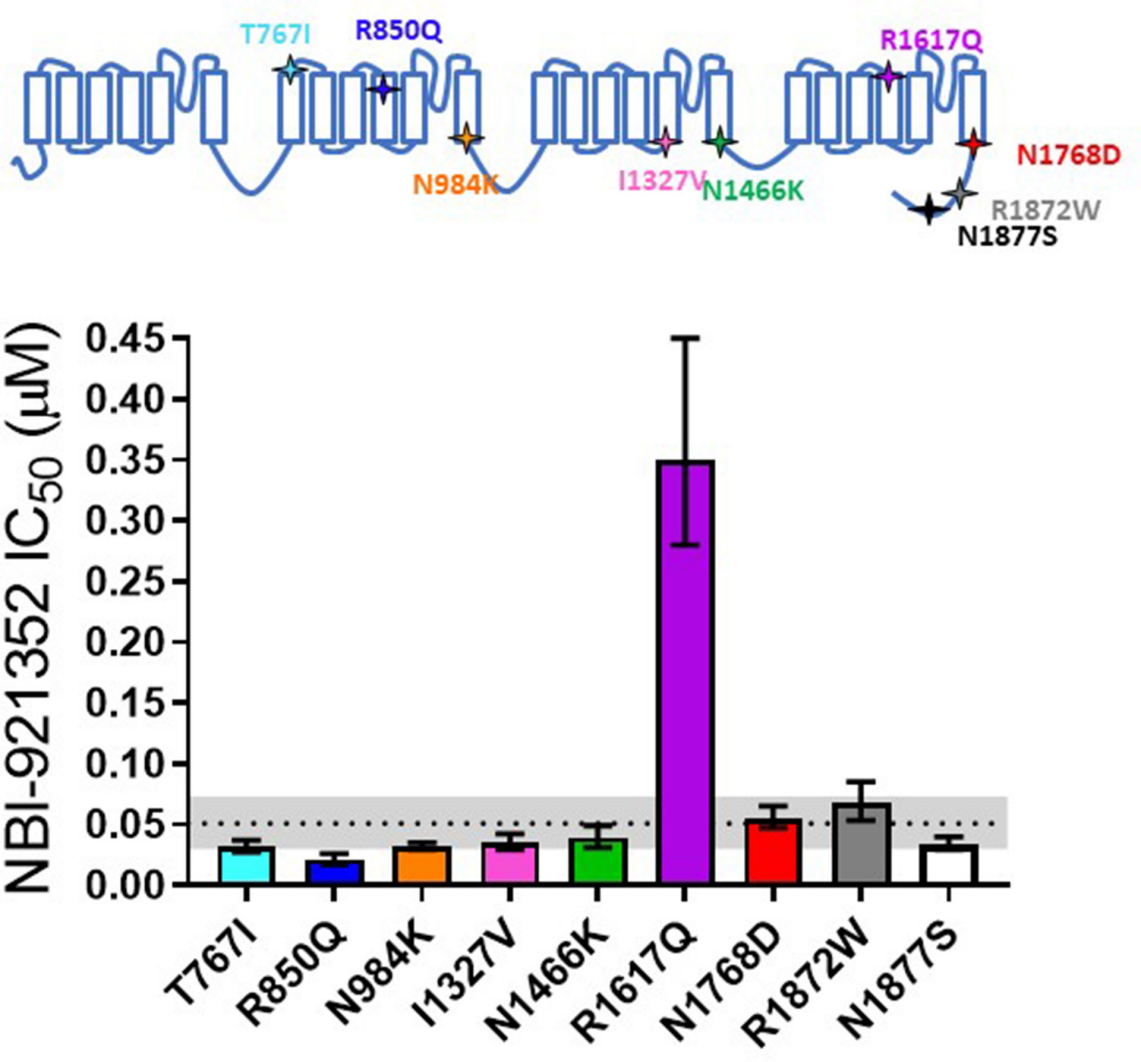
Comparison of NBI-921352 potency on human wild-type Na_V_1.6 and patient-identified variants of Na_V_1.6. All constructs were transiently transfected into Expi293F cells and evaluated by automated patch-clamp electrophysiology using the SophionQube. The voltage-clamp methods were identical to those used for evaluation of the wild-type channels. The error bars indicate the 95% confidence interval of the fitted IC_50_ generated in Prism. The horizontal dotted line is at the IC_50_ for wild type Na_V_1.6 (51 nM, see Figure 1). The gray shaded band indicates the 95% confidence range for the IC_50_ for wild-type Na_V_1.6. Only two variants have fitted IC_50_’s outside of the 95% confidence interval for the wild-type channel IC_50_. R850Q was slightly more potently inhibited and R1617Q was less potently inhibited than the wild-type channel. R1617Q is near the proposed binding site for NBI-921352. Figure 2—source data 1.Quantification inhibition of patient variants.

Patients with *SCN8A*-RES carry missense variants in the Na_V_1.6 channel. A great number of variants have been identified, with a range of biophysical defects. Since most variants are de novo, many have been identified in only one or a few patients. For this reason, we determined the effectiveness of NBI-921352 to inhibit nine patient identified variants spread across the channel (Figure 2, Table 2; Gardella and Møller, 2019; Wagnon and Meisler, 2015). The nine variants studied have all been identified in *SCN8A*-RES patients and are in Domains II, III, IV, and the C-terminus. Inhibition of the mutant channel constructs was evaluated by automated patch-clamp electrophysiological techniques after transient transfection of the human Na_V_1.6 variant construct of interest into Expi293F cells. All the variants were sensitive to inhibition by NBI-921352. Observed IC_50_s for inhibition were 0.051 µM (WT mean from Figure 1), 0.031 µM (95% CI: 0.027–0.037 µM)(T767I), 0.021 µM (95% CI: 0.017–0.026 µM)(R850Q), 0.032 µM (95% CI: 0.029–0.036 µM)(N984K), 0.035 µM (95% CI: 0.029–0.043 µM)(I1327V), 0.039 µM (95% CI: 0.031–0.050 µM)(N1466K), 0.34 µM (95% CI: 0.26–0.44 µM)(R1617Q), 0.055 µM (95% CI: 0.046–0.064 µM)(N1768D), 0.068 µM (95% CI: 0.054–0.085 µM)(R1872W), and 0.035 µM (95% CI: 0.029–0.041 µM)(N1877S). We found that eight of the nine variants were inhibited with a potencysimilar to that of the wild-type channel, with most being slightly more potently inhibited. Only one variant, R1617Q, required markedly higher concentrations of NBI-921352 for inhibition, with an IC_50_for inhibition 6.6-fold higher than that of the wild-type Na_V_1.6 channel. The reduced potency for R1617Qis consistent with the variant residing in the predicted binding site of NBI-921352 in the domain IV voltage sensor, see discussion.

**Table 2.** Comparison of NBI-921352 potency on human wild-type Na_V_1.6 and patient-identified gain-of-function variants of Na_V_1.6. IC_50_s corresponding to Figure 2 are shown in the table and were calculated as indicated for Table 1. The 95% confidence intervals are those determined by the fit of the IC_50_ in Prism.

	WT	T767I	R850Q	N984K	I1327V	N1466K	R1617Q	N1768D	R1872W	N1877S
**hNa_V_1.6 IC_50_ (µM**)	0.051	0.031	**0.021**	0.032	0.035	0.039	**0.349**	0.054	0.067	0.034
**95%** CI	0.030–0.073	0.027–0.037	**0.017–0.026**	0.029–0.035	0.029–0.042	0.031–0.049	**0.28 to** **0.40**	0.047–0.065	0.053–0.085	0.029–0.040
**Fold change** **WT / Variant**	-	0.6	**0.4**	0.6	0.7	0.8	**6.8**	1.1	1.3	0.7

### NBI-921352 is a state-dependent inhibitor

Many small molecule inhibitors of Na_V_ channels bind preferentially to open and or inactivated states (Bean et al., 1983; Courtney et al., 1978; Strichartz, 1976).

Na_V_ inhibitors that are structurally similar to NBI-921352 are known to act by binding to the VSD4 in the ‘UP’, position and hence stabilize inactivated states (Ahuja et al., 2015; McCormack et al., 2013). Charge movement in VSD4 has been linked to the voltage dependence of fast inactivation (Ahern et al., 2016). It is likely that the anionic aryl sulfonamide headgroup of NBI-921352 interacts with the fourth arginine in S4 of VSD4 and prevents return of VSD4 to the rested position and recovery from inactivation as seen for related compounds (Ahuja et al., 2015). For these reasons, we expect that binding will be encouraged by VSD4 residing in the UP, inactivated, state.

We designed our voltage-clamp protocols to encourage high occupancy of inactivated states by holding at depolarized membrane potentials (–45 mV for Na_V_1.1, Na_V_1.2, Na_V_1.3, Na_V_1.4, and Na_V_1.6). The isoforms that inactivate at the most negative membrane potentials (Na_V_1.5 and Na_V_1.7) accumulate excess slow inactivation at –45 mV, reducing the signal size. For these two isoforms, the membrane potential was held at –60 mV to preserve robust assay performance while fully inactivating the channels. Our investigation into the state dependence of NBI-921352 did not attempt to rigorously differentiate binding to open channels versus fast inactivated or fast inactivated versus slow inactivated states.

**Figure 3.**
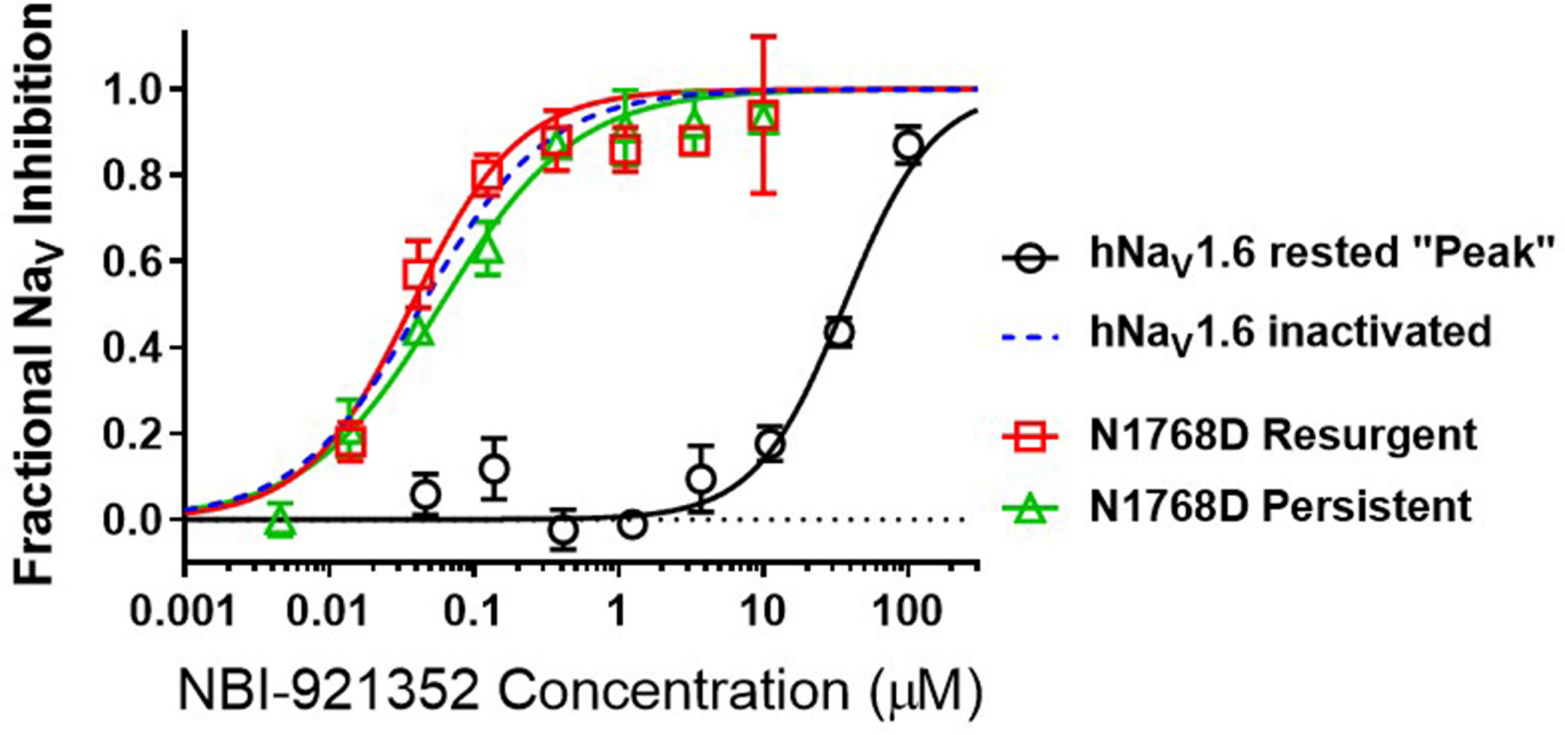
NBI-921352 is a state-dependent inhibitor of Na_V_1.6 and preferentially targets inactivated channels. Concentration-response curves were generated for human WT and N1788D channel isoforms heterologously expressed in HEK293 cells. The analysis included only those cells that met pre-specified acceptance criteria for seal quality, current amplitude, and series resistance. Normalized data from all cell recordings at a concentration were grouped together and plotted with GraphPad Prism 8. Details regarding the number of cells analyzed for each Na_V_ channel isoform and concentration can be found in the source data sheet. Error bars indicating the standard error of the mean fraction were plotted for all points. The blue dotted line indicates the concentration-response curve for wild-type Na_V_1.6 from Figure 1. When Na_V_1.6 channels were equilibrated with NBI-921352 at voltages that allow equilibration with inactivated states (–45 mV), the compound provided potent inhibition, as seen in Figure 1. NBI-921352 also exhibited potent block of Na_V_1.6 when measuring persistent or resurgent sodium current using distinct voltage protocols (see Materials and methods and text). Forcing channels to the rested, closed state by hyperpolarizing to –120 mV resulted in very weak inhibition. Current evoked from very negative potentials is sometimes referred to as ‘peak current’. The 95% confidence intervals for the IC50’s reported in the results are those provided for the error of the fit by Prism. Figure 3—source data 1.Quantification of state dependence of NBI-921352.

To query the state dependence of NBI-921352, we measured the apparent potency with two different voltage protocols that favor either the closed (rested) state or inactivatedstates (Figure 3). Holding the membrane potential at –120 mV induces most channels to reside in the resting state. Brief depolarizations to measure Na_V_1.6 current enabled the determination of an IC_50_of 36 µM (95% CI: 29–47 µM) for rested-state channels. Holding the membrane potential at –45 mV encourages channels to transition into inactivated states. Brief hyperpolarizations allow rapid recovery from inactivation for channels that are not bound to drug followed by a short 20ms test pulse to –20 mV to measure currents from unbound channels (see Materials and methods for details). Measuring the ability of NBI-921352 to inhibit reopening of activated channels leads to an apparent IC_50_ of 0.051 µM (Figures 1 and 3). Thus, NBI-921352 strongly prefers inactivated channels, inhibiting them at concentrations more than 750-fold less than those needed to inhibit rested or ‘peak’ sodium currents.

**Figure 3—figure supplement 1.**
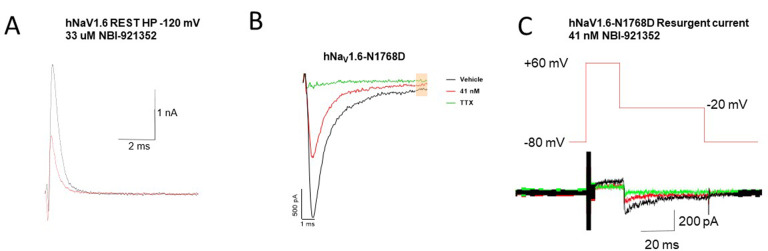
Representative raw traces of the Na_V_1.6 currents used to generate the summary data in Figure 3. (**A**) Currents from WT hNa_V_1.6 channels holding at resting state (voltage = –120 mV) before and after ~IC_50_ concentration of 33 uM NBI-921352 (**B**) The persistent currents of Na_V_1.6-N1768D before and after ~IC_50_ concentration of 41 nM followed by 300 nM of TTX (**C**) Resurgent currents of Na_V_1.6-N1768D before and after ~IC_50_ concentration of 41 nM followed by 300 nM of TTX. Note data archiving/compression led to low resolution of the of trace outside of central region where the peak resurgent currents were measured. Note that in (**A**) currents are outward because the sodium gradients were reversed. This was done to improve assay robustness and reproducibility. See Materials and methods for details.

At more hyperpolarized potentials, potency for all isoforms will tend to be somewhat less. Nonetheless potency on Na_V_1.6 at a more physiologic potential (–62 mV) is shown in Figure 1—figure supplement 2 where the push towards inactivated states is not so strong. NBI-921352 remains potent (IC_50_0.053 µM) in this assay, suggesting that potency and selectivity under physiologic conditions will remain high.

### NBI-921352 inhibited persistent and resurgent currents from mutant Na_V_1.6 channels

The state-dependent nature of inhibition is also revealed in other types of voltage-clamp protocols, including those designed to measure persistent or resurgent sodium currents. Some drugs or candidate drugs, like PRAX-330 and Riluzole, have been touted based on their preference for persistent currents, but this appears to be a feature of many of the compounds in the Na_V_ inhibitor class since they are generally poor inhibitors of closed/rested state channels and bind preferentially to activated channels (Colombo et al., 2013; Mason et al., 2019; Wengert and Patel, 2021). Apparent differences in persistent current selectivity are largely driven by differential kinetics and concentration dependences in combination with the electrophysiological protocols chosen for the measurements.

Elevated persistent and or resurgent currents are believed to underlie or contribute to the pathology of many sodium channel related pathologies (Mason et al., 2019; Pan and Cummins, 2020; Potet et al., 2020; Tidball et al., 2020; Zaman et al., 2019). In most conditions, normal Na_V_1.6 channels inactivate rapidly and nearly completely after opening. Persistent currents result from channels that are not stably inactivated – a common phenotype for epilepsy-inducing variants in Na_V_1.6, including N1768D (Tidball et al., 2020; Wagnon et al., 2015). We found that NBI-921352 inhibited N1768D Na_V_1.6 persistent currents (measured as the non-inactivating current 10ms after initiation of the depolarizing test pulse) with a similar potency as for activated wild-type Na_V_1.6 channels with an IC_50_ of 0.059 µM (95% CI: 0.044–0.082 µM) (Figure 3). A more traditional approach to measuring persistent currents is to step from a very hyperpolarized voltage (for example –120 mV) to a strong depolarization for 50ms or longer. This approach is not viable for NBI-921352 since equilibration of block after from such negative, non-physiological, voltages takes several seconds.

Resurgent currents occur after repolarizing following a strong depolarization as channels redistribute between closed, open, and inactivated states (Raman and Bean, 1997). These resurgent currents are enhanced in many *SCN8A*-RES variants (Pan and Cummins, 2020; Raman et al., 1997). NBI-921352 also effectively inhibited resurgent currents from N1768D channels with apparent IC_50_ of 0.037 µM (95% CI: 0.025–0.060 µM). While the resurgent currents cannot be measured with the same voltage protocols used to measure inactivated state inhibition, these data indicate that N1768D Na_V_1.6 resurgent currents are susceptible to inhibition by NBI-921352 and that inhibition occurs at similar concentrations.

### NBI-921352 preferentially inhibited excitatory pyramidal neurons and spared inhibitory interneurons

**Figure 4.**
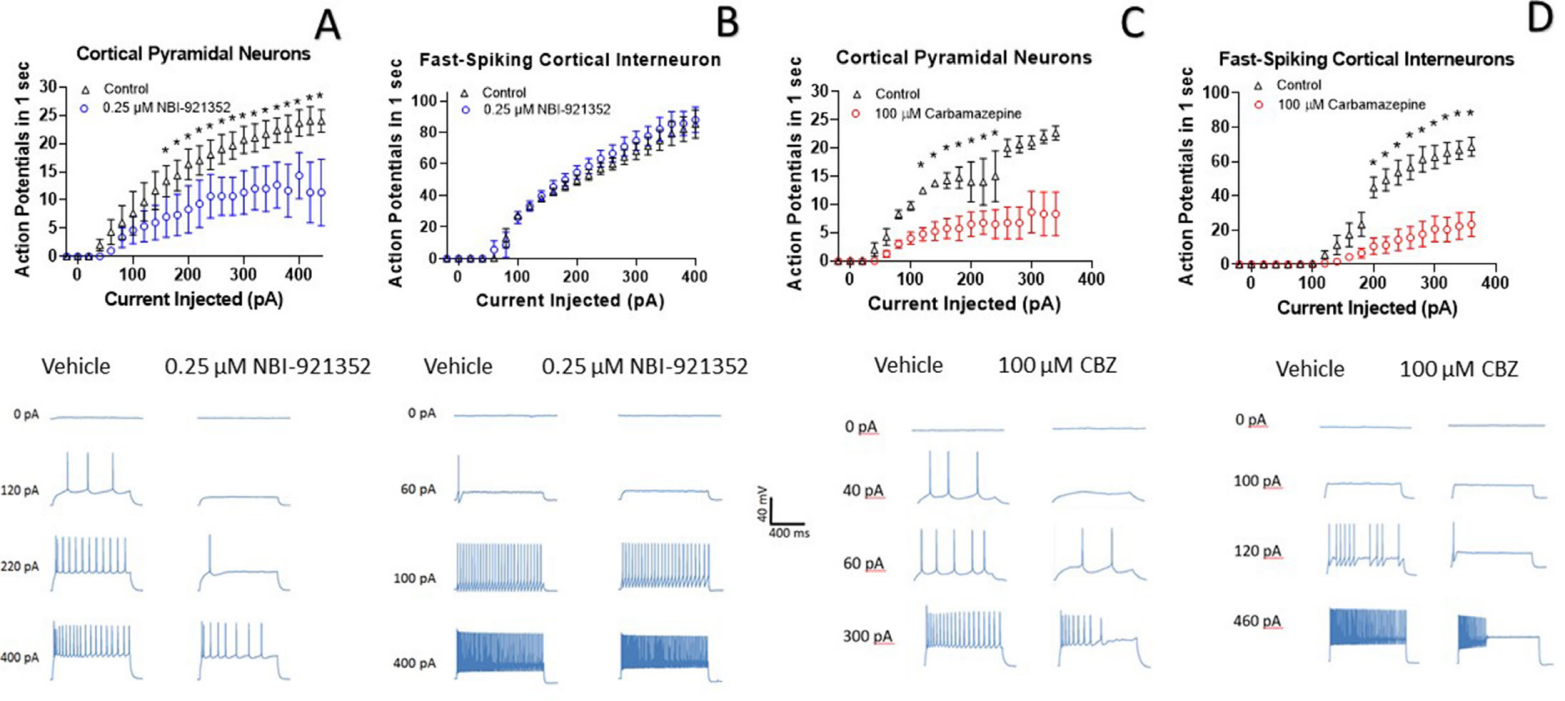
NBI-921352 inhibits firing in pyramidal neurons but spares fast-spiking interneurons. Current input versus action-potential output evaluations in wild-type mouse brain slices treated with vehicle or 0.25 µM NBI-921352 (A & B), or 100 µM carbamazepine (C & D) was plotted. In cortical pyramidal neurons, both NBI-921352 (**A**) and carbamazepine (**C**) reduced action-potential spiking. In fast-spiking cortical interneurons, treatment with NBI-921352 resulted in a trend toward slightly increased firing frequency (**B**), while carbamazepine markedly reduced firing (**D**). The main upper panels compare average action-potential count of 3–4 neurons in each condition±the standard error of the mean. The lower panels show recordings for individual representative neurons for each condition. No inhibitors of synaptic inputs were used for these experiments. The difference in the cumulative area under the curves (AUC) for control versus NBI-921352 treated conditions was evaluated by a paired two tailed t-test (N = 3 or 4 cells for each point). *Indicates a p < 0.05 relative to the control condition. Specific p values are shown in the data transparency Excel file. See Figure 4—figure supplement 1 for more individual neuron comparisons. Figure 4—source data 1.Quantification of cortical neuron current clamp input output.

A primary goal of creating Na_V_1.6 selective inhibitors was to spare Na_V_1.1, the voltage-gated sodium channel that is most prevalent in inhibitory interneurons. This should allow the selective targeting of excitatory neurons, where Na_V_1.6 and Na_V_1.2 are believed to be dominant, over inhibitory interneurons. To test this hypothesis, we performed current-clamp experiments in glutamatergic pyramidal neurons from mouse layer five neocortex and from fast spiking interneurons in the same region. Application of 0.250 µM NBI-921352 decreased the maximum firing rate in all three pyramidal neurons tested (Figure 4A). The difference in the cumulative area under the curves (AUC) for control versus NBI-921352 treated conditions was evaluated by a paired two tailed t-test (N = 3 or 4 cells for each point). Current injection levels > 160 pA led to a significant reduction of the cumulative area under the input output curve (p < 0.05 in a paired two-tailed t-tests) relative to the control condition. Specific p values are shown in the data transparency Excel file. See Figure 4—figure supplement 1 for more individual neuron comparisons.

**Figure 4—figure supplement 1.**
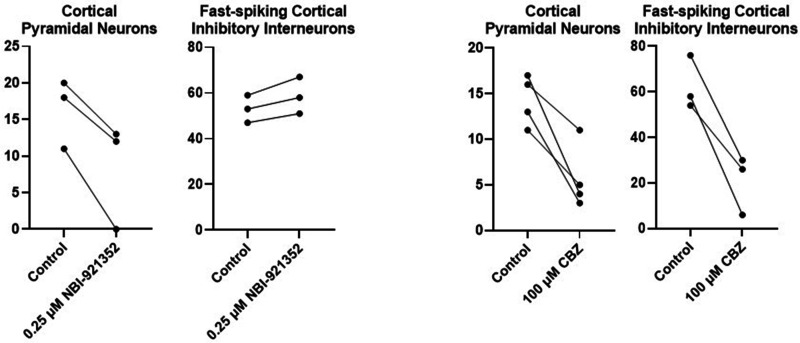
Results for each tested neuron for the neurons summarized in Figure 4. For each neuron, the number of action potentials are shown in response to a current injection level of approximately 3 X the rheobase (determined for each neuron individually) before and after addition of the test compound (NBI-921352 or Carbamazepine). Figure 4—figure supplement 1—source data 1.Quantification of individual neurons.

In contrast, NBI-921352 had no significant effect on the fast-firing inhibitory interneurons tested (Figure 4B). Carbamazepine significantly inhibited action-potential firing in both pyramidal neurons and in fast-spiking interneurons and the degree of inhibition was similar in both types of neurons. A before and after comparison for all tested neurons is shown in Figure 4—figure supplement 1.

### NBI-921352 inhibited electrically induced seizures in *Scn8a^N1768D/+^* mice

A selective inhibitor of Na_V_1.6 should lend itself to the treatment of disease states caused by pathologic gain of function of Na_V_1.6 channels. Hence, we examined the ability of NBI-921352 to inhibit electrically induced seizures in mice with a patient-identified GoF variant in the *Scn8a* gene encoding Na_V_1.6. N1768D is a variant of Na_V_1.6 identified in the first reported *SCN8A*-DEE patient (Veeramah et al., 2012). N1768D Na_V_1.6 channels have impaired voltage-dependent inactivation gating that results in persistent sodium currents and enhanced resurgent currents. Because Na_V_1.6 channels are highly expressed in the neurons of the brain, increased sodium flux in excitatory neurons leads to seizures. Genetically modified mice bearing the same variant (*Scn8a^N1768D/+^*) were created and found to be seizure prone, producing a mouse model with a similar phenotype as that observed in *SCN8A*-DEE patients (Wagnon et al., 2015).Some *Scn8a^N1768D/+^* mice develop spontaneous seizures at age p60 to p100, but seizure onset and frequency is quite variable, making spontaneous seizure studies challenging. In addition, mice rapidly clear NBI-921352, making it extremely difficult to maintain drug plasma and brain levels in an efficacious range for chronic or subchronic dosing experiments. NBI-921352 is more stable in humans with a half-life of elimination of approximately 8.5 hr (Beatch et al., 2020).

As an alternative means of assessing NBI-921352’s ability to engage Na_V_1.6 channels in vivo, we designed a modified version of the 6 Hz psychomotor seizure assay in *Scn8a^N1768D/+^* mice (Barton et al., 2001; Focken et al., 2019). A mild current stimulation (12 mA) evoked robust generalized tonic-clonic seizures (GTC) with hindlimb extensionin *Scn8a^N1768D/+^* mice, but not in wild-type littermates.

**Figure 5.**
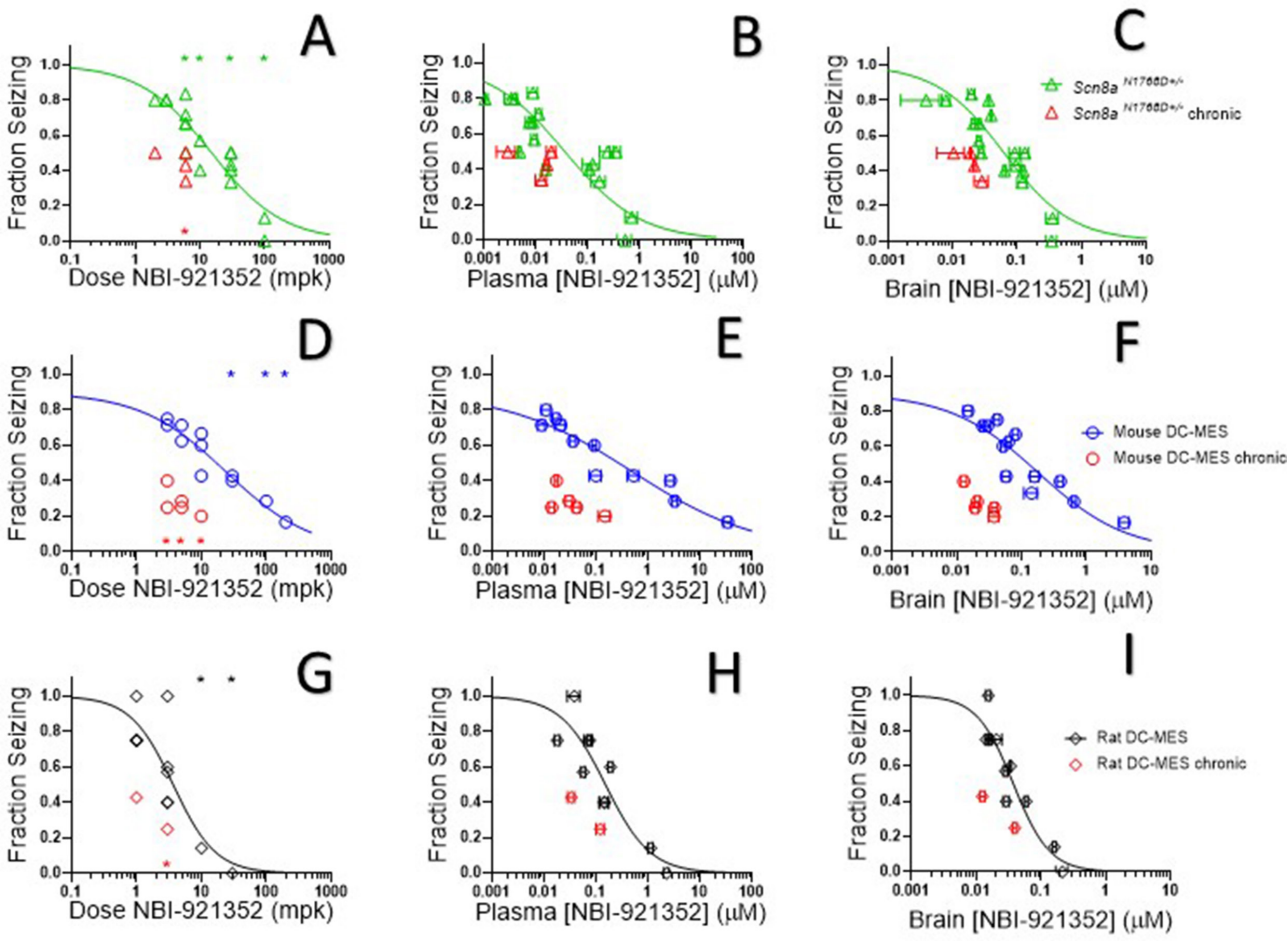
NBI-921352 inhibited electrically induced seizures in rodents. Dose of NBI-921352 is plotted versus efficacy in *Scn8a*^N1768D+/-^ mice in the modified 6 Hz psychomotor seizure assay in A. Plasma concentration of NBI-921352 is plotted versus efficacy in *Scn8a*^N1768D+/-^ mice in the modified 6 Hz psychomotor seizure assay in B. Brain concentration of NBI-921352 is plotted versus efficacy in *Scn8a*^N1768D+/-^ mice in the modified 6 Hz psychomotor seizure assay in C.Green open triangles represent data from animals that received a single dose 2 hr before testing in the seizure assay. Red open triangles represent data from animals that received two daily doses (once every 12 hr) for 6 days. On day 7, these animals were given a final dose (the 13th dosing) 2 hr before testing in the seizure assay. Dose of NBI-921352 is plotted versus efficacy in wild-type mice in the DC-MES assay in D. Plasma concentration of NBI-921352 is plotted versus efficacy inwild-type mice in the DC-MES assay in E. Brain concentration of NBI-921352 is plotted versus efficacy inwild-type mice in the DC-MES assay in F. Blue open circles represent data from animals that received a single dose 2 hr before testing in the seizure assay. Red open circles represent data from animals that received two daily doses (once every 12 hr) for 6 days. On day seven these animals were given a final dose (the 13th dosing) 2 hr before testing in the seizure assay. Dose of NBI-921352 is plotted versus efficacy inwild-type rats in the DC-MES assay in G. Plasma concentration of NBI-921352 is plotted versus efficacy inwild-type rats in the DC-MES assay in H. Brain concentration of NBI-921352 is plotted versus efficacy inwild-type rats in the DC-MES assay in I. Black open diamonds represent data from animals that received a single dose 2 hr before testing in the seizure assay. Red open diamonds represent data from animals that received two daily doses (once every 12 hr) for 6 days. On day 7, these animals were given a final dose (the 13th dose) 2 hr before testing in the seizure assay. Each point represents the fraction of animals exhibiting a GTC with hindlimb extension after stimulus from a dosing group of six to eight animals. Horizontal error bars show the standard error of the mean plasma (**B, E, H**) or brain (**C, F, I**) concentrations measured from the animals in that dosing group immediately after assay. Where error bars are not visible, they are smaller than the symbols. No error bars are shown for the dose levels (**A, D, G**), since those were dictated by the experimenter. Groups receiving the same dose level were combined for statistical analysis. Between-group differences were compared to vehicle response and were analyzed using a Kruskal-Wallis test followed by Dunn’s multiple comparisons test. Statistical significance was reached at values of p < 0.05 and is indicated by the stars at the top of A, D, and G. For specific p values and more details see Figure 5—figure supplement 1 Symbols in red represent animals tested after twice daily dosing for 6.5 days (see description above). Statistical analysis was completed as above for comparison between NBI-921352 treated and vehicle treated groups, and significance is indicated by a red star above the x-axis. In some cases, dose levels that were not statistically significant after a single dose became significant after repeat dosing when compared to vehicle controls. However, in no case were the groups treated with repeatedly with NBI-921352 statistically significant relative to their single dose comparison groups from the same experiment. For more data and p-values see Figure 5—figure supplement 2. Figure 5—source data 1.Quantification of impact of NBI-921352 on rodent seizures.

Oral administration of NBI-921352 2 hr prior to electrical stimulation prevented induction of GTC with hindlimb extension in *Scn8a^N1768D/+^* mice in a dose-dependent manner with a 50% effective dose (ED_50_) of 15 mg/kg (95% CI 9.6–23 mg/kg, see Figure 5A).

**Figure 5—figure supplement 1.**
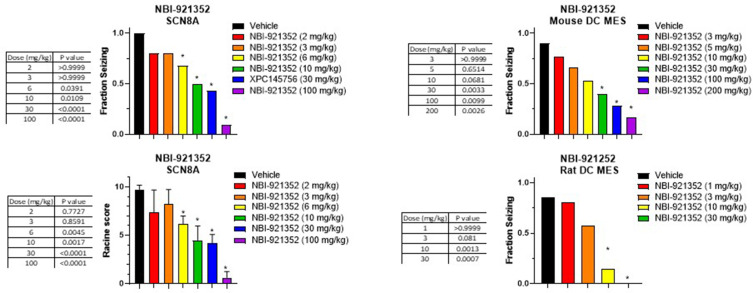
Dose response and statistical analysis of the NBI-921352 dose groups for which brain and plasma concentration response curves are shown in Figure 5. In some cases, multiple experimental groups at the same test dose were assessed. In such cases groups at the same dose level were combined here for comparison to vehicle treated animals. Fraction seizing data is expressed as a mean of the binary seizure score for all tested animals, where animals that seized received a score of 1 and animals that did not seize received a score of 0. In the SCN8A 6 Hz assay two endpoints were noted cumulative Racine score, and presence of a tonic clonic seizure with hind-limb extension. Both scores are presented. Racine score data (bottom left) is expressed as a mean ± SEM. The cumulative racine score evaluation was performed at the same time and in the same animals as the fraction seizing evaluation and provided qualitatively similar results. The Racine score data is not reported in the results section, but we show those values here for comparison. For details see Focken et al., 2019. Between-group differences were analyzed using a Kruskal-Wallis test followed by Dunn’s multiple comparisons test (fraction seizing data) or ordinary one-way ANOVA followed by Dunnett’s multiple comparison test (Racine score data). A statistically significant difference relative to vehicle treated groups is indicated by a star and was reached at values of p < 0.05 (compared to vehicle treated animals). p Values for each comparison to vehicle are shown in the figure.

**Figure 5—figure supplement 2.**
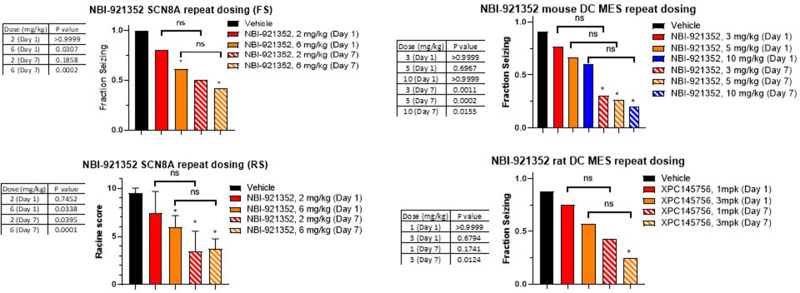
Comparison of single dose versus sub-chronic dosing for NBI-921352. Vehicle, day 1 and day 7 (13 doses BID) were run in parallel with Vehicle animals receiving vehicle every day twice a day. Day 1 animals received 12 doses of vehicle BID before a final dosing with NBI-921352 prior to assay. In some cases, multiple experimental groups at the same test dose were assessed. In such cases groups at the same dose level were combined here for comparison to vehicle treated animals. Fraction seizing data is expressed as a mean of the binary seizure score for all tested animals, where animals that seized received a score of 1 and animals that did not seize received a score of 0. In the SCN8A 6 Hz assay two endpoints were noted cumulative Racine score, and presence of a tonic clonic seizure with hind-limb extension. Both scores are presented. Racine score data (bottom left) is expressed as a mean ± SEM. The cumulative Racine score evaluation was performed at the same time as the fraction seizing evaluation and provided qualitatively similar results. While not reported in the results section we show those values here for comparison. For assay details see Focken et al., 2019. Identification of CNS-Penetrant Aryl Sulfonamides as Isoform-Selective NaV1.6 Inhibitors with Efficacy in Mouse Models of Epilepsy. (*J) Med Chem, 62*(21), 9618–9641. Between-group differences were analyzed using a Kruskal-Wallis test followed by Dunn’s multiple comparisons test (fraction seizing data), or ordinary one-way ANOVA followed by Dunnett’s multiple comparison test (Racine score data). A statistically significant difference relative to vehicle treated groups is indicated by a star and was reached at values of p < 0.05 (compared to vehicle treated animals). p Values for each comparison to vehicle are shown in the figure. In some cases, repeat dosing led to statistically significant protection (relative to vehicle) while its single dose comparator did not. In no case did repeat dosing lead to statistically different efficacy when compared to its same experiment single dose control group (indicated by the comparator bars labeled ‘ns’). For example, in the mouse DC MES assay a single dose of 3, 5, or 10 mg/kg NBI-921352 did not provide statistically significant protection when compared to vehicle (top right panel), but repeated dosing for all three dose levels did protect better than vehicle. However, comparison of repeated doses of 3, 5, or 10 mg/kg were not significantly more protective (p > 0.05) than single doses of the same drug level in the same experiment.

After seizure assessment, all animals were euthanized, and the concentration of NBI-921352 was determined in the plasma and brain tissue from each mouse. The average concentrations for each dose group were used to generate plasma concentration and brain concentration versus efficacy relationships (Figure 5B and C, respectively). The plasma 50% effective concentration (EC_50_) was 0.037 µM (95% CI 0.018–0.090 µM, see Figure 5B). The brain EC_50_ was 0.064 µM (95% CI 0.045–0.091 µM, see Figure 5C).

### NBI-921352 inhibited electrically induced seizures in wild-type mice

Na_V_1.6 is an important mediator of neuronal excitability even in animals without GoFmutations. For this reason, we wondered whether NBI-921352 might have broader application in epilepsy beyond *SCN8A*-RES and in other syndromes of neural hyperexcitability. To gain insight into this possibility, we assessed NBI-921352 in a MES assay induced by direct-current electrical stimulus (DC-MES, see Materials and methods) in wild-type mice. Figure 5D and E & F show that NBI-921352 prevented GTC with hindlimb extension induction in the DC-MES assay in a dose- and concentration-dependent manner. The ED_50_for NBI-921352 was 23 mg/kg (95% CI 16–34 mg/kg, see Figure 5D). The efficacy of NBI-921352 was also concentration dependent with a plasma EC_50_ of 0.52 µM (95% CI 0.25–1.2 µM, see Figure 5E) and a brain EC_50_ of 0.20 µM (95% CI 0.12–0.38 µM, see Figure 5F).

### NBI-921352 inhibited electrically induced seizures in wild-type rats

To further explore the preclinical efficacy of NBI-921352, we assessed NBI-921352 in a MES assay induced by direct-current electrical stimulus in wild-type Sprague Dawley rats (see Materials and methods). Figure 5G & show that NBI-921352 prevented GTC with hindlimb-extension induction in the rat DC-MES assay in a dose- and concentration-dependent manner. The ED_50_ for NBI-921352 was 3.7 mg/kg (95% CI 2.3–7.6 mg/kg, see Figure 5G). The efficacy of NBI-921352 was also concentration dependent with a plasma EC_50_ of 0.15 µM (95% CI 0.09–0.31 µM, see Figure 5H) and a brain EC_50_ of 0.037 µM (95% CI 0.028–0.054 µM, see Figure 5I).

### Repeated-dosing efficacy in mice and rats

We found that repeated dosing tended toincrease efficacy at lower doses and exposures of NBI-921352 than after a single dose (Figure 5, red symbols). Animals were dosed every 12 hr, morning, and evening for 13 doses. Two hours after the 13th dose, on the seventh day, efficacy was tested as in acute-dosing studies. A trend toward improved efficacy was noted in all three assays (see red symbols in Figure 5), but the improvement was not statistically significant when comparing single-dose groups to repeated-dose groups at the same dose level in the same experiment. NBI-921352 did not appreciably accumulate in the plasma or tissue and therefore any trends in improved efficacy were not explained by higher drug concentrations.

### NBI-921352 is effective at lower brain concentrations than three Na_V_ inhibitor ASMs

**Figure 6.**
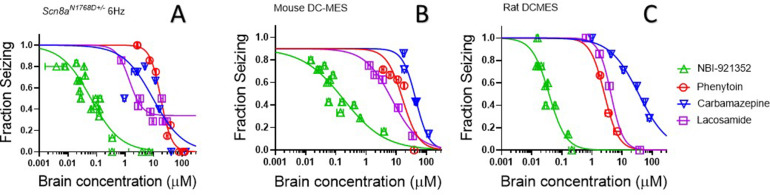
NBI-921352 is more potent than three commonly prescribed Na_V_ inhibitor ASMs. Brain concentration versus fraction of animals exhibiting is plotted for NBI-921352 versus that for phenytoin, carbamazepine, and lacosamide in the *Scn8a^N1768D+/-^* modified 6 Hz model (**A**), the wild-type mouse DC-MES model (**B**), and the wild-type rat DC-MES model (**C**). Each point represents the fraction of animals exhibiting a GTC with hindlimb extension after stimulus from a dosing group of six to eight animals. Horizontal error bars show the standard error of the mean brain concentrations measured from the animals in that dosing group immediately after assay. Where error bars are not visible, they are smaller than the symbols. Statistical analysis of significance of the dose groups for the concentrations shown were performed as in Figure 5 and can be found in Figure 5—figure supplement 1 (NBI-921352), Figure 6—figure supplement 1 (Carbamazepine), Figure 6—figure supplement 2 (Phenytoin), and Figure 6—figure supplement 3 (Lacosamide). Figure 6—source data 1.Quantification of effective brain concentrations of NBI-921352 versus common AEDs.

The efficacy of Na_V_ inhibitors in epilepsy is presumed to bedue to the drugs’ action in the CNS, and CNS side effects are also commonly reported for this class of drugs (Walia et al., 2004). Hence brain concentrations are likely to drive both efficacy and many adverse events. We found that NBI-921352 was effective in the three preclinical seizure models evaluatedat markedly lower brain concentrations than carbamazepine, phenytoin, and lacosamide (Figure 6).The brain EC_50_s for carbamazepine were 9.4 µM, 44 µM, and 36 µM for the *Scn8a^N1768D/+^* 6 Hz model, WT mouse DC-MES, and WT rat DC-MES models, respectively. The brain EC_50_s for phenytoin were 18 µM, 13 µM, and 2.6 µM for the *Scn8a^N1768D/+^* 6 Hz model, WT mouse DC-MES, and WT rat DC-MES models, respectively. The brain EC_50_s for lacosamide were 3.3 µM, 7.1 µM, and 4.3 µM for the *Scn8a^N1768D/+^* 6 Hz model, WT mouse DC-MES, and WT rat DC-MES models, respectively. Thelower brain concentrations required for efficacy with NBI-921352 are consistent with the potent inhibition of Na_V_1.6 produced by NBI-921352 (Figure 1).

**Figure 6—figure supplement 1.**
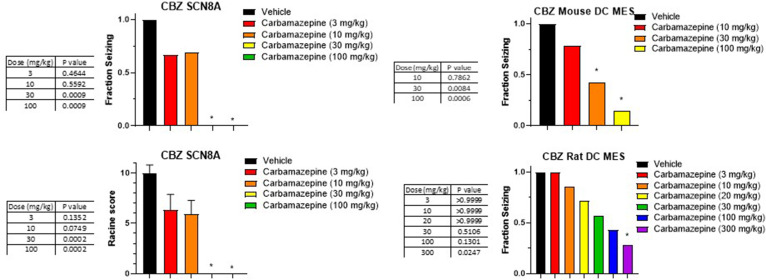
Dose response and statistical analysis of the Carbamazepine dose groups for which brain and plasma concentration response curves are shown in Figures 6 and 7 respectively. In some cases, multiple experimental groups at the same test dose were assessed. In such cases, groups at the same dose level were combined here for comparison to vehicle treated animals. Fraction seizing data is expressed as a mean of the binary seizure score for all tested animals, where animals that seized received a score of 1 and animals that did not seize received a score of 0. In the SCN8A 6 Hz assay two endpoints were noted cumulative Racine score, and presence of a tonic clonic seizure with hind-limb extension. Both scores are presented. Racine score data (bottom left) is expressed as a mean ± SEM. A cumulative Racine score evaluation was performed at the same time as the fraction seizing evaluation and provided qualitatively similar results. While not reported in the results section we show those values here for comparison. For details, see Focken et al., 2019. Identification of CNS-Penetrant Aryl Sulfonamides as Isoform-Selective NaV1.6 Inhibitors with Efficacy in Mouse Models of Epilepsy. (*J) Med Chem, 62*(21), 9618–9641. Between-group differences were analyzed using a Kruskal-Wallis test followed by Dunn’s multiple comparisons test (fraction seizing data) or ordinary one-way ANOVA followed by Dunnett’s multiple comparison test (Racine score data). A statistically significant difference relative to vehicle treated groups is indicated by a star and was reached at values of p < 0.05 (compared to vehicle treated animals). p Values for each comparison to vehicle are shown in the figure.

**Figure 6—figure supplement 2.**
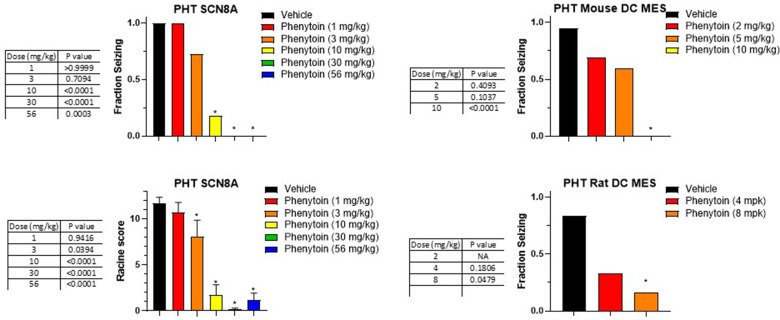
Dose response and statistical analysis of the Phenytoin dose groups for which brain and plasma concentration response curves are shown in Figures 6 and 7 respectively. In some cases, multiple experimental groups at the same test dose were assessed. In such cases groups at the same dose level were combined here for comparison to vehicle treated animals. Fraction seizing data is expressed as a mean of the binary seizure score for all tested animals, where animals that seized received a score of 1 and animals that did not seize received a score of 0. In the SCN8A 6 Hz assay two endpoints were noted cumulative Racine score, and presence of a tonic clonic seizure with hind-limb extension. Both scores are presented. Racine score data (bottom left) is expressed as a mean ± SEM. A cumulative racine score evaluation was performed at the same time as the fraction seizing evaluation and provided qualitatively similar results. While not reported in the results section we show those values here for comparison. For details see Focken et al., 2019. Identification of CNS-Penetrant Aryl Sulfonamides as Isoform-Selective NaV1.6 Inhibitors with Efficacy in Mouse Models of Epilepsy. (*J) Med Chem, 62*(21), 9618–9641. Between-group differences were analyzed using a Kruskal-Wallis test followed by Dunn’s multiple comparisons test (fraction seizing data) or ordinary one-way ANOVA followed by Dunnett’s multiple comparison test (Racine score data). A statistically significant difference relative to vehicle-treated groups is indicated by a star and was reached at values of p < 0.05 (compared to vehicle treated animals). p Values for each comparison to vehicle are shown in the figure.

**Figure 6—figure supplement 3.**
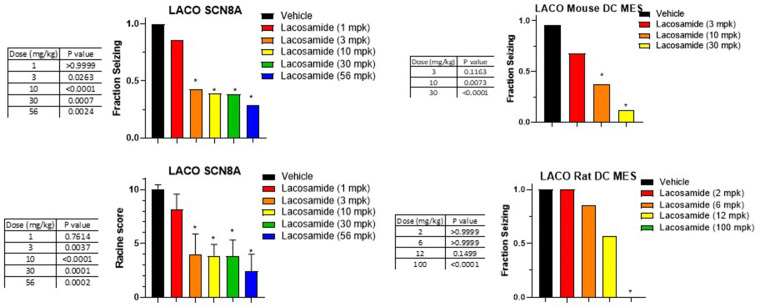
Dose response and statistical analysis of the Lacosamide dose groups for which brain and plasma concentration response curves are shown in Figures 6 and 7 respectively. In some cases, multiple experimental groups at the same test dose were assessed. In such cases groups at the same dose level were combined here for comparison to vehicle-treated animals. Fraction seizing data is expressed as a mean of the binary seizure score for all tested animals, where animals that seized received a score of 1 and animals that did not seize received a score of 0. In the SCN8A 6 Hz assay two endpoints were noted cumulative Racine score, and presence of a tonic clonic seizure with hind-limb extension. Both scores are presented. Racine score data (bottom left) is expressed as a mean ± SEM. A cumulative racine score evaluation was performed at the same time as the fraction seizing evaluation and provided qualitatively similar results. While not reported in the results section we show those values here for comparison. For details see Focken et al., 2019. Identification of CNS-Penetrant Aryl Sulfonamides as Isoform-Selective NaV1.6 Inhibitors with Efficacy in Mouse Models of Epilepsy. (*J) Med Chem, 62*(21), 9618–9641. Between-group differences were analyzed using a Kruskal-Wallis test followed by Dunn’s multiple comparisons test (fraction seizing data) or ordinary one-way ANOVA followed by Dunnett’s multiple comparison test (Racine score data). A statistically significant difference relative to vehicle treated groups is indicated by a star and was reached at values of p < 0.05 (compared to vehicle-treated animals). p Values for each comparison to vehicle are shown in the figure.

### NBI-921352 provided improved separation between efficacy in rats and behavioral signs

The intent of creating a highly selective Na_V_1.6 antagonist was to reproduce or improve on the efficacy of classic, nonselective, sodium channel inhibitor drugs while reducing or preventing the adverse events caused by polypharmacy with other sodium channel and non-sodium channel targets. If sparing Na_V_1.1 and other off-target interactions does, in fact, reduce adverse events, then even higher receptor occupancy of Na_V_1.6 might be achievable, therebyfurther improving efficacy.

**Figure 7.**
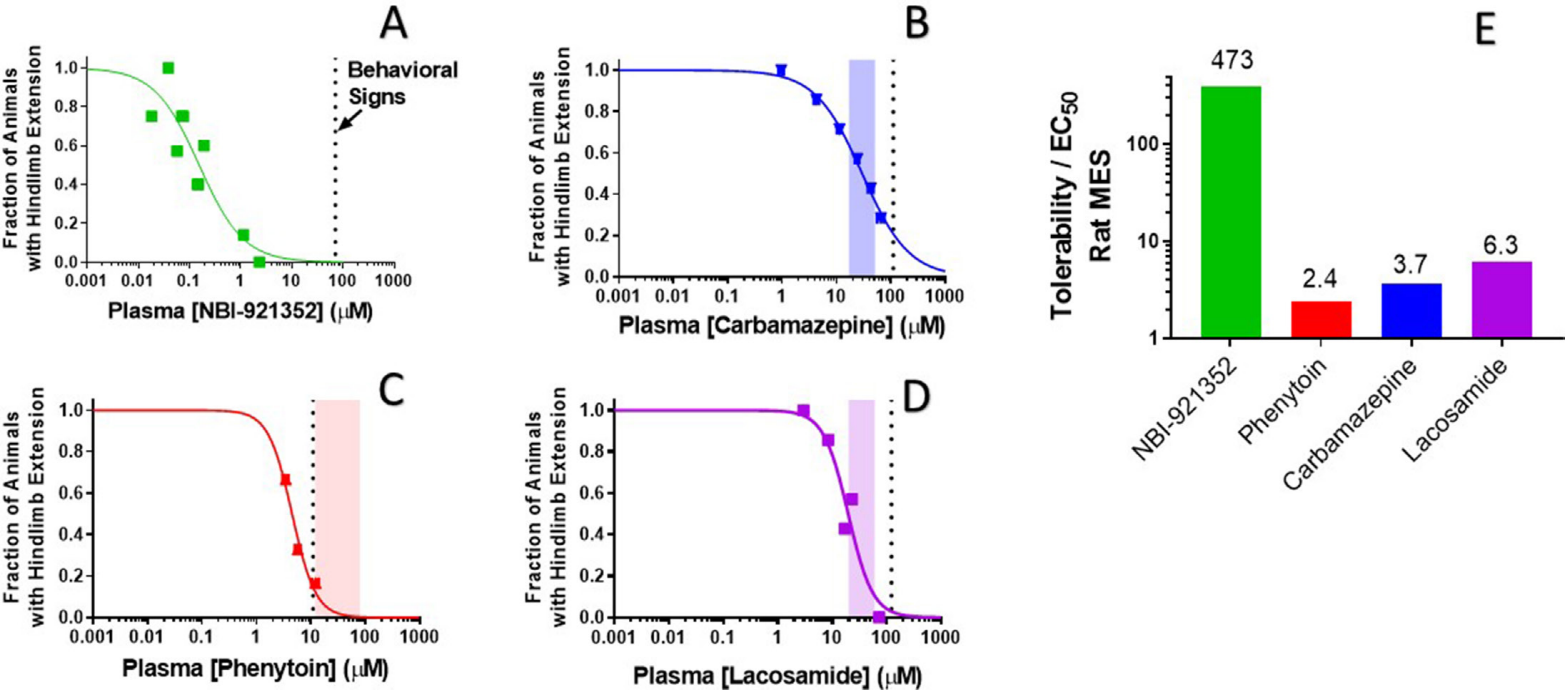
Rat efficacy compared to acute tolerability for NBI-921352 relative to Na_V_ inhibitor ASMs. Plasma concentration versus efficacy data is shown for the rat DC-MES assay for NBI-921352 (**A**), carbamazepine (**B**), phenytoin (**C**), and lacosamide (**D**). The vertical dotted lines indicate the lowest plasma concentration at which a rat was observed to exhibit atypical behavioral signs indicative of an adverse reaction to drug in the assay format. Animals exhibiting such signs were excluded from efficacy evaluation. The shaded bars in B, C, and D indicate the approximate human plasma concentrations observed in clinical practice. Panel E shows the ratio of the (rat plasma EC_50_/ the plasma concentration where behavioral signs were noted for each compound). Statistical analysis of significance of the dose groups for the concentrations shown can be found in Figure 5—figure supplement 1 (NBI-921352), Figure 6—figure supplement 1 (Carbamazepine), Figure 6—figure supplement 2 (Phenytoin), and Figure 6—figure supplement 3 (Lacosamide). Figure 7—source data 1.Quantification of effective and tolerated plasma concentrations of NBI-921352 versus common AEDs.

To evaluate our hypothesis, we compared the window between the plasma concentrations required for efficacy (plasma EC_50_) relative to the minimal plasma concentration at which treated rats showed behavioral signs of adverse effects as reported by the blinded experimenter (Figure 7). We made this comparison both for NBI-921352 and for several widely used Na_V_ inhibitor ASMs: carbamazepine, phenytoin, and lacosamide.

NBI-921352 was well tolerated in these studies up to a plasma concentration of 71 µM. Dividing this concentration by the plasma EC_50_ of 0.15 µMin the rat DC-MES study results in a behavioral signs concentration (BSC) / plasma EC_50_ ratio of 473-fold. The same calculation was repeated for the established ASMs. The minimal plasma concentrations provoking behavioral signs for carbamazepine, phenytoin, and lacosamide were 110 µM, 11 µM, and 123 µM, respectively. Their plasma EC_50_s were 30 µM, 4.5 µM, and 24.4 µM, respectively. Figure 7E shows BSC / Plasma EC_50_ ratios for carbamazepine (3.7-fold), phenytoin (2.4-fold), and lacosamide (5.0-fold). This data indicatesthat increasing Na_V_1.6 selectivity can improve the tolerability of Na_V_ inhibitors in rodent-seizure models.

## Discussion

Sodium channel inhibitors have long been, and remain, a mainstay of pharmacotherapy for epilepsy, as well as for pain and other neurologic, cardiac, and skeletal muscle disorders. The diverse range of indications and systems affected by these drugs is a testament to their critical biological role in cellular excitability. A fundamental challenge for these currently marketed sodium channel drugs is that none of them are selective amongst the nine sodium channel isoforms. As a result, drugs targeting the sodium channels of the brain for epilepsy can inhibit both excitatory and inhibitory neurons, limiting their ability to restore balanced neuronal firing.

Reducing Na_V_1.1 current is proconvulsant due to the predominance of Na_V_1.1 in inhibitory interneurons (Catterall et al., 2010; Claes et al., 2001; Escayg et al., 2000; Mistry et al., 2014; Yu et al., 2006), while the opposite is true for Na_V_1.2 and Na_V_1.6 currents (Ben-Shalom et al., 2017; Hawkins et al., 2011; Martin et al., 2007). Na_V_1.2 and Na_V_1.6 are more highly expressed in excitatory neurons (Catterall et al., 2010; Du et al., 2020; Encinas et al., 2020).

Additionally, these nonselective agents can block the channels associated withskeletal muscle (Na_V_1.4), cardiac tissue (Na_V_1.5), and peripheral neurons (Na_V_1.7, Na_V_1.8, Na_V_1.9). Inhibiting these off-target channels can compromise muscular, cardiovascular,and sensory function. These risks are highlighted by the FDA’s recent drug-safety communication for the nonselective Na_V_ inhibitor ASM lamotrigine. Lamotrigine has been linked to cardiac liabilities as a consequence of Na_V_1.5 inhibition (FDA, 2021). Likewise, Na_V_ inhibitors intended as local anesthetics for trigeminal neuralgia or other pain syndromes and class I cardiac antiarrhythmic drugs are often dose limited by CNS adverse events like dizziness, sedation, and cognitive or motor impairment caused by inhibition of CNS Na_V_ channels (Caron and Libersa, 1997).

An obvious solution to this isoform selectivity challenge is to pursue a precision-medicine approach and create selective pharmacologic agents that preferentially target the sodium channels specific to the desired target tissue or cell type. This selective approach has been pursued for multiple channel isoforms - particularly for the peripheral Na_V_s associated with pain (Na_V_1.3, Na_V_1.7, and Na_V_1.8). This approach has proven challenging because the nine isoforms of sodium channels, Na_V_1.1-Na_V_1.9, share a high degree of primary and tertiary structural similarity. Achieving selectivity with compounds that have tractable pharmaceutical properties has been particularly difficult.

Previous attempts to optimize Na_V_ inhibitors for epilepsy have focused on either drug properties or channel-state dependence. To our knowledge, this is the first description of acentrally penetrant, isoform-selective Na_V_ inhibitor for use in CNS indications, including epilepsy. NBI-921352 represents the first selective inhibitor of Na_V_1.6 that is suitable for systemic oral administration.

The Na_V_1.6 selective profile of NBI-921352 was designed to inhibit activity in excitatory neurons while sparing firing in the inhibitory interneurons where Na_V_1.1 is preferentially expressed. We found that NBI-921352 did reduce firing in cortical excitatory pyramidal cells. In contrast, fast-firing inhibitory interneuron firing was not impaired and actually showed a trend toward increasing (although not statistically significant). The reason that interneuron action-potential firing might increase is unclear but could be a consequence of network effects that arise from interactions with other neurons synapsed onto the target neurons in the experiment. These data confirm that selective Na_V_1.6 inhibitors can display differential inhibition of neurons in a manner that nonselective inhibitors, like carbamazepine, generally do not.

The distribution of Na_V_1.6 to excitatory pyramidal neurons is not absolute, and Na_V_1.6 expression has also been found in inhibitory interneurons. This is most well established for Purkinje neurons (Raman et al., 1997; Woodruff-Pak et al., 2006). Deletion of Na_V_1.6 in Purkinje neurons in mice causes deficits in both cognition and motor control (Levin et al., 2006; Woodruff-Pak et al., 2006). NaV1.6 is also highly expressed at the nodes of Ranvier of both central and peripheral neurons (Boiko et al., 2001; Caldwell et al., 2000; Herzog et al., 2003). We have found that selective inhibitors of Na_V_1.6 can cause ataxia and motor symptoms when plasma and brain concentrations are very high (Figure 7). It is unclear whether these effects are due to concentrations exceeding those that retain high selectivity, or whether high occupancy of Na_V_1.6 is directly responsible. In either case, our data would suggest that selective inhibition of Na_V_1.6 provides a robust window between the level of inhibition required to reduce seizure induction and the levels that impair normal behavior.

*SCN8A*-RES patients most often carry de novo genetic variants. While some variants are known to be recurrent, many variants are represented by a single patient (Meisler, 2019). We therefore wanted to assure that NBI-921352 inhibition was not limited to wild-type Na_V_1.6 channels. We tested nine distinct, patient-identified variants and found that 8 of them were inhibited by NBI-921352 at similar concentrations as wild-type channels (Figure 2). One variant, R1617Q, was found to be 6.8-fold less sensitive to inhibition than the wild-type channel. R1617Q has been identified in multiple *SCN8A*-DEE patients and is in the domain IV voltage-sensor domain (VSD4). NBI-921352 is an aryl sulfonamide with structural similarity to the Na_V_1.7 targeted aryl sulfonamides where the binding site has been identified as the Na_V_1.7 VSD4 (Ahuja et al., 2015; McCormack et al., 2013). It is likely that the R1617Q directly or allosterically impairs the tight association of NBI-921252 with Na_V_1.6 due to its proximity to the binding site. Despite the reduction in potency, NBI-921352 remains markedly more potent than existing Na_V_ inhibitor drugs on the R1617Q variant Na_V_1.6 channel. This would suggest that while NBI-921352 may be an effective treatment for *SCN8A*-DEE patients carrying R1617Q variants, higher plasma levels of the compound could be required for efficacy in those patients.

Many *SCN8A*-RES-associated variants produce their GoF effects by disrupting or destabilizing the inactivation-gating machinery of Na_V_1.6 channels. This can lead to pathological persistent or resurgent currents that contribute to neuronal hyperexcitability (Pan and Cummins, 2020).

Most known small molecule inhibitors of Na_V_ channels, except some marine toxins like tetrodotoxin, bind preferentially to inactivated gating states of the channels and stabilize the channels in inactivated, non-conductive conformations. This state dependence is manifested as a protocol dependence of the apparent drug potency. State dependence inhibition has been described in many ways. Use-dependent, frequency-dependent, resurgent current -selective, and persistent current-selective inhibition are all consequences of a preference for binding to inactivated channels. Stabilizing inactivated states of the channel reduces persistent and resurgent currents, and this feature has been suggested to contribute to the efficacy of many Na_V_ targeted ASMs including phenytoin, carbamazepine, oxcarbazepine, lacosamide, cannabidiol, and lamotrigine (Wengert and Patel, 2021).NBI-921352 is also highly state dependent, with a > 750 fold preference for inactivated channels vs. rested, closed-state channels (sometimes referred to as *peak current*). Forcing all Na_V_1.6 channels into the closed state by applying voltages more hyperpolarized than physiological (–120 mV) results in very weak inhibition of the channels (Figure 3). Biasing the channels toward inactivated states by holding the membrane potential more positive in a protocol designed to monitor currents recovered from the inactivated state, resurgent current, or persistent current protocol all yielded potent inhibition. In physiologic conditions, channels are distributed among closed, open, and inactivated states, thus allowing equilibration of potent inhibition of the channel by NBI-921352.

Based on structural similarities and biophysical behavior, we believe that NBI-921352 binds to the domain IV voltage sensor domain (VSD4), much like GX-936 binds to the chimeric NaV1.7 constructs in Ahuja et al. In this structure the VSD4 is captured in the up (activated) state. The down (rested) state of the voltage sensor is not available for high-affinity interaction with the compound. We expect that the high affinity binding events for NBI-921352 occur only when the VSD4 is in the up state, promoting inactivation. We believe this is the likely mechanism by which NBI-921352 traps channels in the inactivated state and reduces Na_V_1.6 currents.

Increasing the selectivity of a Na_V_ inhibitor provides the expectation of an improved safety profile by reducing adverse events caused by off-target activity. An inherent risk of this approach is the potential loss of efficacy that could come from reduced polypharmacy. We have developed a potent, highly selective Na_V_1.6 inhibitor in NBI-921352. Our studies with NBI-921352 indicate that a Na_V_1.6-specific compound can retain a robust ability to prevent seizures in rodent models at modest plasma and brain concentrations, consistent with the important role of Na_V_1.6 in seizure pathways. Our data also suggests that this selectivity profile does improve the tolerability of NBI-921352 relative to commonly employed nonselective sodium channel ASMs in rodents. Whether these results will translate to humans is not yet established, but Phase I clinical trials have shown that NBI-921352was well tolerated at plasma concentrations higher than were required for efficacy in the preclinical rodent studies described here. NBI-921352 is currently being developed for both *SCN8A*-DEE epilepsy and adult focal-onset seizures by Neurocrine, 2019. Phase II clinical trials will soon evaluate the efficacy of NBI-921352 in patients (Neurocrine, 2021). These clinical trials will provide the first evidence for whether the robust efficacy and tolerability demonstrated in rodents translates to human epilepsy patients.

## Materials and methods

**Key resources table:** 

Reagent type (species) or resource	Designation	Source or reference	Identifiers	Additional information
Cell line (*H. sapiens*)	Expi293F	Thermo Fischer	cat# A14527	SCN8A mutant transient transfections
Cell line (*H. sapiens*)	FreeStyle 293 F	Thermo Fischer	cat# R710-07	Stable transfections
Strain, strain background (*M. musculus*)	C57BL/6 J male Scn8a^N1768D/+^ (stock#400690)x C3HeB/FeJ female (strain#000658).Both Male and Female heterozygous Scn8a^N1768D/+^ micewere tested	Licensed from Miriam Meisler, Univ. of Michigan.Wagnon et al., 2015		Colony maintained at The Jackson Laboratory
Strain, strain background (*M. musculus*)	CF-1, male	Charles River	Code: 023	
Strain, strain background(*R. norvegicus*)	Sprague Dawley, male	Envigo	Code: 002	
Chemical compound, drug	NBI-921352	US Patent #10246453 B2	Compound ID #101	Synthesized at Xenon Pharmaceuticals
Chemical compound, drug	Carbamazepine	Sigma-Aldrich	C4024	
Chemical compound, drug	Phenytoin	Sigma-Aldrich	D4505	
Chemical compound, drug	Lacosamide	Toronto Research Chemicals	L098500	
Gene (*H. sapiens*)	SCN1A	GenBank	NM_006920	
Gene (*H. sapiens*)	SCN2A	GenBank	NM_021007	
Gene (*H. sapiens*)	SCN3A	GenBank	NM_0069220	
Gene (*H. sapiens*)	SCN4A	GenBank	NM_000334	
Gene (*H. sapiens*)	SCN5A	GenBank	NM_198056	
Gene (*H. sapiens*)	SCN8A	GenBank	NM_014191	
Gene (*H. sapiens*)	SCN9A	GenBank	NM_002977	
Gene (*M. musculus*)	SCN1A	GenBank	NM_018733.2	
Gene (*M. musculus*)	SCN2A	GenBank	NP_001092768.1	
Gene (*M. musculus*)	SCN8A	GenBank	NM_001077499	
Gene (*H. sapiens*)	SCN1B	GenBank	NM_199037	
Gene (*H. sapiens*)	FGF13	GenBank	NM_033642	
Gene (*H. sapiens*)	CNTN1	GenBank	NM_001843	
Recombinant DNA reagent	pcDNA4/TO (vector)	Thermo Fischer	cat #V102020	Vector for SCNxA genes
Recombinant DNA reagent	pcDNA6/TR(regulatory vector for tetracycline repressor protein)	Thermo Fischer	cat#V102520	Vector to generate inducible FreeStyle 293 F and Expi293F
Recombinant DNA reagent	pcDNA3.1 (+)(vector)	Thermo Fischer	cat#V79020	Vector for SCN1B gene
Recombinant DNA reagent	pcDNA3.1/Hygro(+)	Thermo Fischer	cat# V87020	Vector for FGF13 and CNTN1 genes

### Electrophysiological determination of potency and selectivity

#### Cell lines

Electrophysiology experiments were performed with HEK293 cells either stably transfected or transiently transfected. All of the cell lines tested negative for mycoplasma. The cell lines were authenticated by STR profiling at EuroFins Genomics.Stable cell lines were transfected with an expression vector containing the full-length cDNA coding for specific human and mouse sodium channel α-subunit, grown in culture media containing 10% fetal bovine serum, and 0.5 mg/mL Geneticin (G418) at 37 °C with 5% CO_2_. The Na_V_1.x stable cell lines and accessory constructs used correspond to the following GenBank accession numbers: Human Na_V_1.1 (NM_006920); mouse Na_V_1.1 (NM_018733.2); human Na_V_1.2 (NM_021007); mouse Na_V_1.2 (NP_001092768.1); human Na_V_1.5 (NM_198056); human Na_V_1.6 (NM_014191); mouse Na_V_1.6 (NM_001077499); human Na_V_1.7 (NM_002977); human Na_V_1.4 (NM_000334); human Na_V_1.3 (NM_0069220). The human Na_V_ β1 subunit (NM_199037) was co-expressed in all cell lines. Human and mouse Na_V_1.6 channels were also coexpressed with human FHF2B (NM_033642) to increase functional expression. Human Na_V_1.2 channels were also coexpressed with Contactin 1 (NM_001843) to increase functional expression.

For studies of mutant channels, cDNA plasmids in pcDNA4/TO Mammalian Expression Vector were transiently transfected into Expi293F cells stably expressing human FHF2b and human SCN1B subunit (polyclonal) background using ExpiFectamine 293 Transfection Kits (Gibco,Thermo Fisher Scientific CAT #: A14524). Induction was achieved using Tetracycline (Sigma Aldrich). Transfected cells were used in automated patch-clamp experiments 24 hours postinduction.

#### Na_V_ channel automated Qube 384 planar patch-clamp assays

NBI-921352 requires several seconds to equilibrate with activated channels, and this property of the compound must be taken into consideration in the design of state-dependent assay voltage-clamp protocols.

Data was collected using the Qube 384 (Sophion) automated voltage-clamp platform using single hole plates. To measure inactivated state inhibition, the membrane potential was maintained at a voltage where inactivation is complete. For each Na_V_ channel subtype, the V_h_used to quantify compound inhibition were as follows: Na_V_1.6 (-45 mV), Na_V_1.1 (-45 mV), Na_V_1.2 (-45 mV), Na_V_1.3 (-45 mV), Na_V_1.5 (-60 mV), Na_V_1.7 (-60 mV), Na_V_1.4 (-45 mV). The mutant channel hNa_V_1.6^N1768D^ was found to have accelerated run-down compared with wild-type hNa_V_1.6, so the holding potential was adjusted to –60 mV to provide sufficient signal window. The voltage was briefly repolarized to a negative voltage (–150 mV) for 20 ms for Na_V_1.5, Na_V_1.7, Na_V_1.3, Na_V_1.4or for 60 ms for Na_V_1.1, Na_V_1.2, and Na_V_1.6 to allow recovery from fast inactivation, followed by a test pulse to –20 or 0 mV for 10 ms to quantify the compound inhibition. The repolarization step allows compound-free channels to recover from fast inactivation, but compound-bound channels remain inhibited during the subsequent test step. For rested state ‘Peak’ current V_h_was set to –120 mV. Appropriate filters for minimum seal resistance were applied (typically >500 MΩ membrane resistance), and series resistance was compensated at 100%. The pulse protocols were run at 1 Hz for hNa_V_1.7, hNa_V_1.5, hNa_V_1.3, and hNa_V_1.4 or 0.04 Hz for Na_V_1.6, Na_V_1.1 and Na_V_1.2.

To construct concentration response curves, baseline currents were established after 20 min in vehicle (0.5% DMSO). Full inhibition response amplitudes were determined by adding tetrodotoxin (TTX, 300 nM) or tetracaine for Na_V_1.5 (10 µM) to each well at the end of the experiment. Compounds were then exposed at a single concentration for 20 min. One-sixth of every experimental plate was dedicated to vehicle-only wells that enabled correction for nonspecific drift (i.e. rundown) of the signal in each experiment. For all channel subtypes, inhibition by the compound reached steady state within 20 min of incubation. The current inhibition values (I_(CPD)_) were normalized to both the vehicle (I_control_) and the full response defined by supramaximal TTX (I_TTX_) or tetracaine (for Na_V_1.5) addition responses according to Equation 1.

$$I_{norm(CPD)}=(I_{CPD}-I_{control})/(I_{TTX}-I_{control}).$$

This normalized inhibition was then further normalized to the span of the assay to account for the run-down seen in cells exposed to vehicle alone for 20 min as follows:

Equation 2

$$I_{norm,span}=(I_{norm(CPD)}-I_{norm(VEH)})/(1-I_{norm(VEH)}),where:$$

*I_norm, span_* = the current response normalized to within the span of the assay.
*I_norm(CPD)_* = the normalized response in the presence of compound.
*I_norm(VEH)_* = the normalized response in the absence of compound.

This normalization ensures that the data ranges were between 0 and 1, and there is no rundown in the plots. The normalized data from all cell recordings at a concentration were grouped together and plotted with GraphPad Prism 8, and IC_50_ values were calculated for grouped data using the following version of the Hill equation:

Equation 3

$$Y=RD+(1-RD)*[CPD]/(IC_{50}+[CPD]),where:$$

*Y* = the fraction of sodium current blocked in the presence of the compound.
*[CPD]* = the concentration of compound.
*IC_50_* = the IC_50_ concentration.

RD = the ‘rundown’ of sodium current in vehicle alone, which is equal to 0 in this case, as the inhibition has already been normalized to the span.

The Hill slope was fixed to 1.
The 95% CI for the IC_50_ from the fitted curve to the mean data were reported unless otherwise noted.

To evaluate inhibition of hNa_V_1.6(N1768D) resurgent currents, synthetic Na_V_β4 peptide (KKLITFILKKTREKKKECLV) was added to the intracellular recording solution at 200 μM and a dedicated protocol to elicit resurgent currents was employed (Barbosa et al., 2015). Cells were voltage clamped at (V_h_ = –80 mV) and subjected to a strong depolarization ( + 60 mV) for 20 ms. Following the strong depolarization, cells were partially repolarized to the voltage where resurgent current was maximal (–20 mV) for 50 ms, and resurgent current amplitude was measured. This resurgent current-specific waveform was repeated at 5 Hz for 100 s in vehicle, followed by 100 s in test compound, then 300 nM TTX. Fractional inhibition was calculated using the same normalization procedure as above.

Experiments were all performed at 27°C ± 2°C.

#### Automated patch-clamp recording solutions

The recording solutions for Na_V_1.1, Na_V_1.2, Na_V_1.3, Na_V_1.4 and Na_V_1.6 cell line studies contained: Intracellular solution (ICS): 5 mM NaCl, 10 mMCsCl, 120 mMCsF, 0.1 mM CaCl_2_, 2 mM MgCl_2_, 10 mM HEPES (4-(2-hydroxyethyl)–1-piperazineethanesulfonic acid buffer), 10 mM EGTA (ethylene glycol tetraacetic acid); adjusted to pH 7.2 with CsOH. Extracellular solution (ECS): 140 mM NaCl, 5 mMKCl, 2 mM CaCl_2_, 1 mM MgCl_2_, 10 mM HEPES; adjusted to pH 7.4 with NaOH. Solutions with a reversed Na^+^ gradient were used for Na_V_1.5 and Na_V_1.7 studies since they improved technical success. ICS: 120 mM NaF, 10 mMCsCl, 0.1 mM CaCl_2_, 2 mM MgCl_2_, 10 mM HEPES, 10 mM EGTA; adjusted to pH 7.2 with CsOH. ECS: 1 mM NaCl, 139 mMCholineCl, 5 mMKCl, 2 mM CaCl_2_, 1 mM MgCl_2_, 10 mM HEPES; adjusted to pH 7.4 with NaOH. Osmolarity in all ICS and ECS solutions was adjusted with glucose to 300 mOsm/kg and 310 mOsm/kg, respectively.

### Current-clamp recording of cortical pyramidal neurons and inhibitory interneurons

#### Slice preparation

Parasagittal cortical brain slices were prepared from> P21 mice using standard procedures (adapted from Tai et al., PNAS 2014). Briefly, the mouse was deeply anaesthetized with isoflurane and decapitated. The brain was removed and placed into chilled artificial cerebrospinal fluid (aCSF) solution containing (in mM): 125 NaCl, 25 NaHCO3, 2.5 KCl, 1.25 NaH_2_PO_4_, 2 CaCl_2_, 2 MgCl_2_, 10 d-glucose, pH 7.3, osmolarity adjusted to ~306 mOsm using sucrose. All solutions were saturated with 95% O_2_ and 5% CO_2_ constantly perfused with 95% O_2_/5% CO_2_. Slices with a thickness of 400 µm were prepared using a vibratome (Ted Pella, Inc). Following sectioning, the slices were placed in a holding chamber and incubated in a water bath at 34 °C for 15 min. The brain slices were removed from the water bath and held at room temperature for 60 min prior to recording.

#### Brain slice electrophysiology assay

All experiments involving rodent subjects were performed in accordance with the guidelines of the Canadian Council on Animal Care (CCAC). Following a 60-min incubation at room temperature, a brain slice was selected and placed on the stage of an upright microscope (SliceScope Pro 2000, Scientifica). The slice was constantly perfused with room temperature aCSF, containing 0.1% DMSO as a vehicle control, and oxygenated with 95% O_2_/5% CO_2_.The slice was visualized using brightfield microscopy, and a healthy neuron was selected from neocortical layer 5. Whole-cell configuration was achieved with a pipette (bath resistance 4–6 MΩ) containing internal solution. Stimulation was applied in current-clamp mode, and consisted of a series of 1000ms square pulses, beginning at –20 pA and increasing by +20 pA increments (3000ms between pulses).

Once the recordings in vehicle were completed, and while still holding the patch on the same neuron, the bath solution was changed from 0.1% DMSO in aCSF to 0.25 µM NBI-921352 or 100 µM Carbamazepine in aCSF. The slice was incubated in circulating compound for 10 min before repeating the series of square pulse stimulations. Working stock solutions were prepared in DMSO at a concentration of 20 mM.

All data analysis was done offline using ClampFit 10.7 (Molecular Devices). Data are presented as a mean ± SEM. For each sweep, the number of evoked APs was counted, and plotted as a function of current injection (beginning with –20 pA). These generated ‘input/output’ (or ‘F/I’) curves demonstrating the relationship between stimulus and average AP frequency. Statistical significance was assessed using paired, two-way, student’s t-test applied at each current injection level with significance considered p < 0.05.

### Formulation and oral dosing of NBI-921352

#### Vehicle preparation

The vehicle for oral dosing solutions was 0.5% methyl cellulose and 0.2% Tween-80 in deionized (DI) water. DI water (0.8 L) was heated up to 70–80°C. Five grams of methyl cellulose was slowly added to heated DI water. The mixture was stirred until it formed a homogeneous milky suspension. The suspension was moved to a cold room and stirred overnight to get a clear solution. Two milliliters of Tween-80 was added to the clear solution and diluted up to 1 L with DI water. The vehicle solution was stored at 2–8°C.

#### Drug formulation

NBI-921352 was weighed into vials. An appropriate amount of vehicle was added to the NBI-921352 powder then mixed on a T18 ULTRA TURRAX homogenizer (IKA, Wilmington, NC) to create a uniform suspension at the desired concentration. The vials were then wrapped in aluminum foil to protect them from light and placed on a stir plate until the time of dosing. Carbamazepine and lacosamide were formulated in the same manner.Phenytoin was formulated in 0.9% physiological saline.

#### Dosing

NBI-921352, carbamazepine, and lacosamide were administered orally using a stainless-steel gavage needle at a dose volume of 10 ml/kg. Phenytoin was formulated in physiologic saline and was administered intraperitoneally (i.p.) using a 25-gauge needle at a dose volume of 10 mL/kg. All compounds were administered 2 hr prior to electrical seizure induction for all seizure models employed in this study.

### Bioanalytical assessment of plasma and brain concentrations

#### Sample collection:

Approximately 0.5 mL of blood was collected from each mouse at the end of the assay via cardiac puncture under deep anesthesia. The blood samples were collected in a syringe and transferred to tubes containing EDTA. Blood was stored at 4 °C until centrifuged within 30 min of collection. Plasma was harvested and placed on dry ice and stored in a freezer set to maintain a temperature of –70°C to –80°C until analysis. Brains were harvested immediately after blood collection and placed on dry ice prior to storage in a freezer set to maintain a temperature of –70°C to –80°C until analysis.

#### Plasma samples:

Extraction of plasma samples was carried out by protein precipitation using acetonitrile. Plasma samples (50 µL) were mixed with 50 µL of internal standard (IS) solution in water followed by addition of 10 µL of concentrated ortho-phosphoric acid and 200 µL of acetonitrile. Samples were vortexed for 30 seconds, centrifuged at 13,000 rpm for 20 min, decanted in to a 96-well plate, and further centrifuged at 4000 rpm for 20 min. The samples were analyzed by UHPLC-ESI-MS/MS as described below.

#### Brain samples:

Prior to extraction, pre-weighed whole brains were homogenized in 1:1 acetonitrile/water (v/v) (4 mL per mouse brain) using an IKA T18 ULTRA-TURRAX Homogenizer at the setting of 4 for approximately 2 min. The homogenate was centrifuged at 13,000 rpm for 20 min and 50 µL of the supernatant were treated exactly as described above for plasma samples. Fifty µL of the brain homogenate were then treated exactly as the plasma samples described above.

#### Standards and quality control (QC) samples:

K_2_EDTA Blank mouse plasma purchased from Valley Biomedical, California, USA was used to prepare standards and QC samples for plasma quantitation and as surrogates for brain homogenate quantitation. Calibration samples ranged from 2.34 ng/mL to 4800 ng/mL. QC samples concentration included 14 ng/mL (QC-L), 255 ng/mL (QC-M), and 3600 ng/mL (QC-H). Standards and QC samples were processed the same way as the sample extracts described above.

#### Analytical methods and statistics for plasma and tissue samples:

Samples were analyzed by UHPLC-ESI MS/MS using a TQ-5500 Sciex triple quadrupole mass spectrometer equipped with a Shimadzu Nexera UHPLC pump and auto-sampler system using an ACE C18 PFP, 2.50 × 50 mm, 1.7 µm particle size column and gradient elution consisting of solvent A (0.1% formic acid in water) and solvent B (0.1% formic acid in acetonitrile) starting at 20% B from 0 min to 0.4 min and then increased to 100% B from 0.4 min to 0.6 min. At 2.0 min, the mobile phase composition was switched back to 60% B for 1 min. The flow rate used throughout the experiment was 0.4 min/mL. The analyte, NBI-921352, and the IS were detected by electrospray in the positive ion mode using the following transitions: m/z 460/91 for NBI-921352 and m/z 503/341 m/z for the IS. The UHPLC-ESI MS/MS system was controlled by Analyst 1.6.

Sample concentrations were determined using a linear calibration function, weighted 1 /X, generated by the regression of analyte to IS peak area ratios in the standard samples to their respective concentrations. Acceptance criteria for the analytical run required that the back calculated values of the standards and the QC samples fell within ±20% of their nominal values, except for the lowest standard or lower limit of quantitation (LLOQ), for which the acceptance criterion was ±25%. At least 6 out of 12 standard points had to show back-calculated values within ±20% of their nominal concentrations for the calibration to be accepted. At least three QC samples, one at each level, had to show back-calculated values within ±20% of their nominal concentrations for the whole sample batch to be valid.

### Animals

After delivery, animals were allowed sufficient time to acclimate prior to testing (~1 week). All animals were housed in plastic cages in rooms with controlled humidity, ventilation, and lighting (12 hr/12 hr light–dark cycle). All animal procedures were performed using protocols approved by Xenon Animal Care Committee and the Canadian Council on Animal Care.

#### Scn8a^N1768D/+^ mice:

Xenon Pharmaceuticals Inc licensed the mouse with the missense mutation p.Asn1768Asp (N1768D) in the neuronal sodium channel Na_V_1.6, characterized and developed by Dr. M Meisler (University Of Michigan, MI, USA). The *Scn8a^N1768D^* knock-in allele was generated by TALEN targeting of (C57BL/6JXSJL) F2 eggs at the University of Michigan Transgenic Animal Model Core. The line was propagated by backcrossing N1768D/+ heterozygotes to C57BL/6 J wild-type mice (The Jackson Laboratory, Bar Harbor, ME). Male N1768D/+ heterozygotes on a C57BL/6 J background were subsequently backcrossed to C3HeB/FeJ female mice. All the experiments were performed using animals following at least seven such backcrosses. Experiments were performed using (B6× C3 He) F7 (F7. N1768D/+) offspring aged 35–42 days.

#### WT mice:

Adult male CF-1 WT albino mice 26–35 g were obtained from Charles River, Senneville, Quebec, Canada. All the assays were carried out in mice 9–12 weeks of age.

#### Sprague-Dawley rats:

Adult male Sprague-Dawley albino rats weighing 150–200 g were obtained from Envigo, Livermore, CA, USA. All the assays were carried out in rats aged 5–6 weeks.

### The modified 6 hz psychomotor seizure assay

*Scn8a^N1768D/+^*heterozygous mice were tested at 5 weeks of age (p35). The modified 6 Hz seizure assay in *Scn8a^N1768D/+^*heterozygous mice was adapted from the traditional 6 Hz assay psychomotor seizure assay to provide a measure of in vivo on target (Na_V_1.6 mediated) efficacy (Barton et al., 2001). The modified assay used a low frequency (6 Hz) but long-duration stimulation (3 s) to induce seizures. We identified 12 mA and a 0.3-ms pulse interval as a suitable current for testing in *Scn8a^N1768D/+^* mice, since it differentiated mutant and wild-type (WT) mice. An electroshock (6 Hz, 12 mA) was delivered for 3 s (at 0.3-ms pulse interval) by corneal electrodes (Electro Convulsive Therapy Unit 57,800 from Ugo Basile). Immediately prior to the electroshock, the animals’ eyes were anesthetized with a drop of Alcaine (0.5% proparacaine hydrochloride). Upon corneal stimulation, WT mice experienced mild seizure behaviors such as facial clonus, forelimb clonus, Straub tail, rearing, and falling, but did not experience a generalized tonic-clonic seizure (GTC) with hindlimb extension. *Scn8a^N1768D/+^* animals, however, in addition to mild seizure behaviors, experienced a GTC with hindlimb extension. The modified assay showed a clear differentiation of seizure behavior between WT and *Scn8a^N1768D/+^* mice. *Scn8a^N1768D/+^* mice exhibited GTC with hindlimb extension but not WT mice.

For the single-dose and repeated-dose efficacy experiments, *Scn8a^N1768D/+^* animals were dosed PO with vehicle or NBI-921352 two hours before the administration of the electric stimulation. An animal was considered protected in the assay upon prevention of GTC with hindlimb extension and was then scored ‘0’. An animal displaying GTC with hindlimb extension was considered not protected and is then scored ‘1’. The experimenter scoring the seizure behavior was blinded to the treatment.

### DC-Maximal electroshock seizure assay in rodents

The maximal electroshock seizure (MES) assay has been extensively used in the search for anticonvulsant substances (Löscher et al., 1991; Piredda et al., 1985; White et al., 1995). The MES assay is sensitive to nonselective NaV inhibitors. It is considered a model for generalized tonic-clonic (GTC) seizures and provides an assessment of seizure spread. Briefly, an electroshock of direct current (DC) was delivered by corneal electrodes (Electro Convulsive Therapy Unit 57,800 from Ugo Basile). The parameters of stimulation were different between mice and rats. In CF1 mice aged 9–12 weeks, a direct current of 50 mA (60 Hz) was delivered for 0.2 s (pulse width of 0.5ms), whereas in Sprague Dawley (SD) rats aged 5–6 weeks, a direct current of 150 mA (60 Hz) was delivered for 0.3 s (pulse width of 0.5ms). Immediately prior to the electroshock, the animals’ eyes were anesthetized with a drop of Alcaine (0.5% proparacaine hydrochloride). Upon corneal stimulation, naive animals experienced a generalized tonic-clonic seizure (GTC) with hindlimb extension.

For the efficacy experiments, single dose and repeated dose, animals were dosed PO with vehicle or NBI-921352 2 hr before the administration of the electric stimulation. An animal was considered protected in the assay in the absence of a GTC with hindlimb extension and is then scored ‘0’. An animal displaying GTC with hindlimb extension was considered not protected and is then scored ‘1’. The experimenter scoring the seizure behavior was blinded to the treatment.

### Blinding of in vivo efficacy experiments

On each testing day, individual treatment groups were assigned a random label (e.g. A, B, C, etc.) by the technical staff administering the compound. To ensure blinding, the technical staff member performing drug administration differed from the person performing the test. Therefore, the experimenter conducting testing was blinded to treatment group (e.g. drug or vehicle treatment, dose, and time point).

### Randomization of in vivo efficacy experiments

Randomization of animals into various treatment groups occurred on a per-animal (e.g. rather than a per-cage) basis. Therefore, each animal was randomly assigned to a treatment group, and all animals tested in each experiment had an equal chance of assignment to any treatment group. Prior to each study, a randomization sequence was obtained (https://www.graphpad.com/quickcalcs/).

### Observation of potential adverse behavior for Figure 7

Animals were observed by the scientist(s) responsible for dosing for the first 30 min after dosing for and again at the time of assay (2 hr post dose) for signs of abnormal behavior. Abnormal behavioral signs were noted for all compounds – tremor, ataxia and hypoactivity – when plasma concentrations were sufficiently high. The lowest concentration at which such behavioral signs were noted in any of the animals tested is illustrated by the dotted line in Figure 7. Animals that exhibited abnormal behavior were not assessed in efficacy assays.

### Data processing and analysis

All statistics were calculated using GraphPad Prism version eight software. All fraction seizing data plotted by dose groups is expressed and is an absolute fraction of all tested animals that seized (number of animals that seized / the number of animals tested). Racine score data is reported in Figure 5—figure supplements 1 and 2, and in Figure 6—figure supplements 1 and 2, and 3. Racine score data are plotted by dose groups and are expressed as mean ± SEM. Between-group differences were compared to vehicle response and were analyzed using a Kruskal-Wallis test followed by Dunn’s multiple comparisons test (fraction seizing data) or ordinary one-way ANOVA followed by Dunnett’s multiple comparison test (Racine score data). Statistical significance was reached at values of p < 0.05. More detailed results of the statistical analysis, with p values, is presented in the figure supplements and legends.

## Data Availability

All the numerical data used to generate the figures in contained in the excel data source file included in the submission.

## Additional files

Transparent reporting form
