# Supplementary material for: NBI-921352, a first-in-class, NaV1.6 selective, sodium channel inhibitor that prevents seizures in Scn8a gain-of-function mice, and wild-type mice and rats
Source: eLife. 2022 Mar 2;11:e72468. doi: 10.7554/eLife.72468 (PMC8903829; doi:10.7554/eLife.72468)
Supplement: Transparent reporting form [file elife-72468-transrepform1.pdf]

Westbrook Centre
Milton Road
Cambridge CB4 1YG, UK
P 01223 855340
W elifesciences.org
T @elife

eLife's transparent reporting form

We encourage authors to provide detailed information *within their submission* to facilitate the interpretation and replication of experiments. Authors can upload supporting documentation to indicate the use of appropriate reporting guidelines for health-related research (see EQUATOR Network), life science research (see the BioSharing Information Resource), or the ARRIVE guidelines for reporting work involving animal research. Where applicable, authors should refer to any relevant reporting standards documents in this form.

If you have any questions, please consult our Journal Policies and/or contact us: editorial@elifesciences.org.

Sample-size estimation

- You should state whether an appropriate sample size was computed when the study was being designed
- You should state the statistical method of sample size computation and any required assumptions
- If no explicit power analysis was used, you should describe how you decided what sample (replicate) size (number) to use

Please outline where this information can be found within the submission (e.g., sections or figure legends), or explain why this information doesn't apply to your submission:

> We did not perform formal power calculations for in vitro and in vivo experiments, but used generally accepted sample sizes depending on the type of experiment and our past experience. Sample size is denoted in figure legends, the accompanying Excel data file, and in some cases, in the Results section. For in vivo experiments only data points with n≥ 4 were included.

Replicates

- You should report how often each experiment was performed
- You should include a definition of biological versus technical replication
- The data obtained should be provided and sufficient information should be provided to indicate the number of independent biological and/or technical replicates
- If you encountered any outliers, you should describe how these were handled
- Criteria for exclusion/inclusion of data should be clearly stated
- High-throughput sequence data should be uploaded before submission, with a private link for reviewers provided (these are available from both GEO and ArrayExpress)

Please outline where this information can be found within the submission (e.g., sections or figure legends), or explain why this information doesn't apply to your submission:

> The number of experiments is denoted by animal sample size, numbers of cells, or number of assay replicates. All replicates are defined as biologic replicates, in that distinct animals/cells/wells were analysed once. No outliers were identified or excluded as part of the in vivo or ex vivo datasets. For automated electrophysiology experiments, the analysis included only those cells that met pre-specified acceptance criteria for seal quality, current size and series resistance. More detailed information can be found in figure legends, the accompanying Excel data file, and in the Results and Materials and Methods sections.

eLife Sciences Publications, Ltd is a limited liability non-profit non-stock corporation incorporated in the State of Delaware, USA, with company number 5030732, and is registered in the UK with company number FC030576 and branch number BR015634 at the address Westbrook Centre, Milton Road, Cambridge CB4 1YG.

Statistical reporting

- Statistical analysis methods should be described and justified
- Raw data should be presented in figures whenever informative to do so (typically when N per group is less than 10)
- For each experiment, you should identify the statistical tests used, exact values of N, definitions of center, methods of multiple test correction, and dispersion and precision measures (e.g., mean, median, SD, SEM, confidence intervals; and, for the major substantive results, a measure of effect size (e.g., Pearson's r, Cohen's d)
- Report exact p-values wherever possible alongside the summary statistics and 95% confidence intervals. These should be reported for all key questions and not only when the p-value is less than 0.05.

Please outline where this information can be found within the submission (e.g., sections or figure legends), or explain why this information doesn't apply to your submission:

> All statistical methods are described in detail in the Material and Methods section. Information on data presentation is provided in the figure legends as space allowed and the complete datasets (including N) can be found in the accompanying Excel data file. The statistical test for each experiment is described in the figure legend, along with values for N, mean, error (SEM, STD or confident intervals based on type of experiment and statistical test used) and p-values where $p<0.05$. No corrections for multiple testing were required.

(For large datasets, or papers with a very large number of statistical tests, you may upload a single table file with tests, Ns, etc., with reference to sections in the manuscript.)

Group allocation

- Indicate how samples were allocated into experimental groups (in the case of clinical studies, please specify allocation to treatment method); if randomization was used, please also state if restricted randomization was applied
- Indicate if masking was used during group allocation, data collection and/or data analysis

Please outline where this information can be found within the submission (e.g., sections or figure legends), or explain why this information doesn't apply to your submission:

> As described in the Materials and Methods sections regarding in vivo experiments, the technical staff member performing drug administration differed from the person performing the test. On each testing day, individual treatment groups were assigned a random label (e.g., A, B, C, etc.) by the technical staff administering the compound. Therefore, the experimenter conducting testing was blinded to treatment group (e.g., drug or vehicle treatment, dose, and time point). Randomization of animals into various treatment groups occurred on a per-animal (e.g., rather than a per-cage) basis. Therefore, each animal was randomly assigned to a treatment group, and all animals tested in each experiment had an equal chance of assignment to any treatment group. Prior to each study, a randomization sequence was obtained (www.graphpad.com/quickcalcs).

Additional data files ("source data")

- We encourage you to upload relevant additional data files, such as numerical data that are represented as a graph in a figure, or as a summary table

eLife Sciences Publications, Ltd is a limited liability non-profit non-stock corporation incorporated in the State of Delaware, USA, with company number 5030732, and is registered in the UK with company number FC030576 and branch number BR015634 at the address Westbrook Centre, Milton Road, Cambridge CB4 1YG.

- Where provided, these should be in the most useful format, and they can be uploaded as "Source data" files linked to a main figure or table
- Include model definition files including the full list of parameters used
- Include code used for data analysis (e.g., R, MatLab)
- Avoid stating that data files are "available upon request"

Please indicate the figures or tables for which source data files have been provided:

Source data can be found in the accompanying Excel data file for all Tables and Figures.

eLife Sciences Publications, Ltd is a limited liability non-profit non-stock corporation incorporated in the State of Delaware, USA, with company number 5030732, and is registered in the UK with company number FC030576 and branch number BR015634 at the address Westbrook Centre, Milton Road, Cambridge CB4 1YG.
