# Supplementary material for: NBI-921352, a first-in-class, NaV1.6 selective, sodium channel inhibitor that prevents seizures in Scn8a gain-of-function mice, and wild-type mice and rats
Source: eLife. 2022 Mar 2;11:e72468. doi: 10.7554/eLife.72468 (PMC8903829; doi:10.7554/eLife.72468)
Supplement: Peer review history

# Editor report: Editor's evaluation

**DOI:** 10.7554/eLife.72468.sa0

**Authors:** Baron Chanda

**Affiliations:** https://ror.org/01yc7t268 Washington University in St. Louis United States

This exciting study reports on the characterization of a novel compound that preferentially targets Nav1.6 voltage-gated sodium channels and shows substantial activity against epilepsy associated SCN8A mutations and seizure activity in a variety of animal models. This compound and approach has significant promise to be translated into a therapeutic for individuals with treatment resistant epilepsy.

# Decision letter

**DOI:** 10.7554/eLife.72468.sa1

**Affiliations:** https://ror.org/01yc7t268 Washington University in St. Louis United States

Our editorial process produces two outputs: (i) public reviews designed to be posted alongside the preprint for the benefit of readers; (ii) feedback on the manuscript for the authors, including requests for revisions, shown below. We also include an acceptance summary that explains what the editors found interesting or important about the work.

**Decision letter after peer review:**

Thank you for submitting your article "NBI-921352, a First-in-Class, Na V 1.6 Selective, Sodium Channel Inhibitor That Prevents Seizures in Scn8a Gain-of- Function Mice, and Wild-Type Mice and Rats" for consideration by *eLife*. Your article has been reviewed by 2 peer reviewers, and the evaluation has been overseen by a Reviewing Editor and Richard Aldrich as the Senior Editor. The following individual involved in review of your submission has agreed to reveal their identity: Theodore Cummins (Reviewer #2).

The reviewers have discussed their reviews with one another, and the Reviewing Editor has drafted this to help you prepare a revised submission. Please note that some of the points noted below are related and should be addressed comprehensively.

Essential revisions:

1) The compound was not tested against Nav1.8 and Nav1.9 channels. It may be unlikely that NBI-921352 would inhibit either of these as they are more divergent in the likely binding region than the other isoforms test, this is not a given and is a slight weakness. While testing these two isoforms is not impossible, it may not be necessary. But the lack of data at least needs to be discussed.

2) To facilitate the binding/action of NBI-921352, a depolarized holding potential (-45 mV) was used in the voltage-clamp recording for most VGSC isoforms (except Nav1.7 and Nav1.5) and Nav1.6 variants (except N1768D). This EP protocol was used for Nav1.1, Nav1.2, and Nav1.6, the three major VGSC isoforms in the central nervous system, and therefore, the comparison among those three isoforms should be reliable, despite the depolarized holding potential (-45 mV). However, it is unlikely for neurons to experience such sustained depolarizing state (-45 mV) under physiological conditions, and therefore, the selectivity of NBI-921352 on Nav1.6 vs other VGSCs under physiological conditions could be different compared to the values reported here. It is better to hold cells at physiologically relevant membrane potentials or using action potential waveforms derived from real AP recordings of pyramidal neurons or interneurons. The authors should discuss these limitations, and possible impact on their assessment of selectivity against other VGSCs in their native cellular backgrounds.

3) While the electrophysiological data clearly show that NBI-921352 shows voltage-dependence and likely state dependence, the study lacks an assay of use-dependence using repetitive higher frequency depolarizations. Something like 10 or 20 Hz pulsing might be informative.

4) In several places it is stated that NBI-921352 preferentially inhibits activated (inactivated or open) channels. There is no direct assay looking at inhibition of open but not-inactivated channels, so this conclusion is potentially misleading. The authors could try something like a WCW non-inactivating construct, but I think just rewording those places where it says inactivated or open to maybe "inactivating and possibly also open" would be reasonable.

5) Different voltage-clamp protocols were adopted among different sodium channel isoforms (lines 406-418) to measure IC50 concentrations of the drug, which requires clarification/justification. Since NBI-921352 is "a state-dependent inhibitor" (line 30) and "requires several seconds to equilibrate with activated channels" (line 401), the difference in holding potentials (-60 mV vs -45 mV), recovery period (20 ms vs 60 ms at -150 mV), and data acquisition rates (1 Hz vs 0.04 Hz) may alter the binding/interaction of NBI-921352 with tested channels, and therefore, introduce bias in the comparison of selectivity among VGSCs. The authors should explain their rationale for choosing these protocols and address the potential confounding effects in data analysis.

6) The statement that a preference for persistent currents is, "in fact, a feature of all the compounds in the Nav inhibitor class" is not justified by either data presented in the study or by an appropriate reference. The "in fact" is particularly not justified since it really states a bias that would need to be bolstered by hard data. Not much in biology is proven as a fact. This bold statement may have some validity, but it is not really important to the major conclusions of the study.

7) It is stated that NBI-921352 is rapidly cleared in mice, but it is unclear if this is different in humans, which could limit the translational potential. It would help the reader if this was discussed.

8) Lines 248-249 states unequivocally that the efficacy and adverse events of Nav inhibitors is driven by action in the CNS. There is no citation for this and it comes across as another bias. It seems intuitive that the anti-seizure activity is dependent on CNS activity, but this is not demonstrated in the current study. Adverse events could involve sodium channels in muscle and/or the peripheral nervous system. Therefore this statement should be modified.

9) Details on what behaviors were monitored and how they were monitored for figure 7 need to be included or else the bars and discussion on this aspect of the study should be removed.

10) Nav1.6 is highly expressed in Purkinje neurons and motor neurons, and plays important roles in motor system. Did the authors observe any motor impairment in the behavior studies? It would have been informative to examine the effect of NBI-921352 on AP firing and resurgent currents in Purkinje neurons. As Nav1.6 is the major isoform found at nodes of Ranvier in myelinated fibers, including motor neurons axons, it is critical to know if NBI-921352 alters motor neuron and/or motor activity.

11) Three seizure models were used to evaluate the effect of NBI-921352 on electrically induced seizure. The data shows that NBI-921352 prevented the GTC with hindlimb extension in all three assays, demonstrating its potential to become an antiepileptic drug. In an earlier work from the same group (J Med Chem 2019; 62: 9618-9641), a modified Racine scale (0-5) was used to score the severity of different seizure activities, while only GTC with hindlimb extension (score 5 in Racine scale) is considered as seizure in this study. Is there any reason to choose this evaluation criteria instead of Racine scale used in previous work?

12) The authors tested seizure behaviors after repeated dosing of the drug, but claimed that there was no statistically significant difference between single-dose group vs repeated-dose group (lines 238-246). I was unable to find the description of statistical analysis method for behavioral studies in the manuscript.

13) There are several problems in the brain slice recording experiments. First, paired student t-test was used to compare the AP firing before and after drug treatment, which is inappropriate. Repeated measures two-way ANOVA should be used here, or use area under curve rather than number of APs for statistical analysis. Second, despite the dominant effect of Nav1.1 in inhibitory interneurons, Nav1.6 is also expressed in interneurons, and to confirm selectivity in the mouse model, it is better to examine the effect of NBI-921352 on AP firing in Nav1.6-knockout neurons. Third, the experiments lack a vehicle control data set. Fourth, the number of cells for each group is low (3-4 cells), more recordings should be performed.

14) Please add a section of statistical analysis in Materials and methods, and list the statistical analysis method used in each experiment.

[Editors' note: further revisions were suggested prior to acceptance, as described below.]

Thank you for resubmitting your work entitled "NBI-921352, a First-in-Class, Na V 1.6 Selective, Sodium Channel Inhibitor That Prevents Seizures in Scn8a Gain-of- Function Mice, and Wild-Type Mice and Rats" for further consideration by *eLife*. Your revised article has been evaluated by Richard Aldrich (Senior Editor) and a Reviewing Editor.

The manuscript has been improved but there are some remaining issues that need to be addressed, as outlined below:

1. The reviewer 1 pointed out the lack of NBI-921352 data on Nav1.8 and Nav1.9, and the authors performed limited study on hNav1.8 co-expressed with β3 in CHO cells.

I am not clear why the authors selected CHO cells as the expression system, as it is known that expression of Nav1.8 is poor in CHO and HEK293 cells, even in the presence of β subunits (Nature 2002, 417: 653-656). The ND7/23 is a more commonly used system for Nav1.8 expression (for example, Toxins 2021, 13:501). I have obtained decent Nav1.8 currents (over 10 nA, with properties similar to those measured in DRG neurons) in ND7/23 cells, but barely saw Nav1.8 currents in HEK 293 cells co-transfected with hNav1.8 and β3. It is unclear how good the Nav1.8 expression was in CHO cells in authors' hands, the authors should include raw traces of Nav1.8 in Figure 1-Supplement 3 to demonstrate the reliable Nav1.8 expression in this assay. If the expression of Nav1.8 is not good, I would recommend removing the Nav1.8 data, though the authors still need to explain the lack of data on those two isoforms and discuss possible effect of NBI-921352.

Typos: there are quite some typos in figure legend of Figure 1-Supplement 3. For example, line 1045, "% Block (Tonic) = (1 ITPI,NBI921352 / ITPl,Control) X 100%", should "ITPl" be "ITP1"? And the calculation should be "(1-ITP1,NBI921352/ITP1,Control) x 100%"? Same typo is shown in line 1046.

In line 1049, "% Block (inactivation state) -", should "-" be "="?

2. I have concerns on different holding potentials being used in determining IC50 of NBI-921352 for different isoforms. The authors responded that "We chose to focus on what we refer to as "molecular selectivity," the fundamental ability for a compound to bind to the channel and stabilize the high affinity conformation. We accomplish this by choosing voltages that promote the same fraction of channels to be in the high affinity (inactivated) state." I wonder how the authors determined that "-45 mV" is the potential for NBI-921352 to bind and reach the high affinity conformation of Nav1.1, 1.2, 1.3, 1.4, and 1.6, and "-60 mV" for Nav1.7 and Nav1.5, there is no justification of this in the manuscript. In addition, when a medicine is applied to a patient, the adverse effect is determined by its affinity and effect on all targeted channels/molecules physiological or pathological conditions, therefore, the relative selectivity of NBI-921352 determined using the same protocol/condition would be more meaningful for clinical practice.

The new data that NBI-921352 showed similar potency in Nav1.6 channel at -62 mV when compared to that at -45 mV is important, this data suggests that the IC50 of NBI-921352 is not significantly changed by the holding potential (or membrane potential) between -62 mV and -45 mV. Although I don't agree with the authors' explanation, I will not ask for extra experiments. However, unless the authors provide convincing evidence that the two holding potentials (-45 mV and -60 mV) used in this study do allow different isoforms to be in their "highest affinity state (inactivated)", the explanation in Page 6 (line 124 to line 138) needs to be modified/removed.

Typo: line 137, "is this assay" should be "in this assay".

3. Regarding to the state of channels that NBI-921352 preferentially inhibits, the abstract wrote "NBI-921352 is a state-dependent inhibitor, preferentially inhibiting inactivated channels", while the figure legend 3 shows "NBI-921352 is a state-dependent inhibitor of NaV1.6 and preferentially inhibited activated (open or inactivated) channels". In main text (page 20, line 424-426), "NBI-921352 is also highly state dependent, with a >750-fold preference for activated channels vs. rested, closed-state channels (sometimes referred to as peak current)". Which state does NBI-921352 preferentially act?

4. The figure legend 5 is confusing (page 63, line 1159-1169), please rephrase the sentences.

# Reply: Author response

**DOI:** 10.7554/eLife.72468.sa2

Essential revisions:

1) The compound was not tested against Nav1.8 and Nav1.9 channels. It may be unlikely that NBI-921352 would inhibit either of these as they are more divergent in the likely binding region than the other isoforms test, this is not a given and is a slight weakness. While testing these two isoforms is not impossible, it may not be necessary. But the lack of data at least needs to be discussed.

Response: We have a limited dataset on inhibition of hNa_V_1.8, and we have added this data to the Results section, and to the table in Supplementary file 1. We do not have ready access to an hNa_V_1.9 assay (internally or commercially). Since neither Na_V_1.8 or Na_V_1.9 are believed to be involved in epilepsy or central excitability we chose not to pursue additional data. We have now discussed this gap in the text. See Results line 108.

2) To facilitate the binding/action of NBI-921352, a depolarized holding potential (-45 mV) was used in the voltage-clamp recording for most VGSC isoforms (except Nav1.7 and Nav1.5) and Nav1.6 variants (except N1768D). This EP protocol was used for Nav1.1, Nav1.2, and Nav1.6, the three major VGSC isoforms in the central nervous system, and therefore, the comparison among those three isoforms should be reliable, despite the depolarized holding potential (-45 mV). However, it is unlikely for neurons to experience such sustained depolarizing state (-45 mV) under physiological conditions, and therefore, the selectivity of NBI-921352 on Nav1.6 vs other VGSCs under physiological conditions could be different compared to the values reported here. It is better to hold cells at physiologically-relevant membrane potentials or using action potential waveforms derived from real AP recordings of pyramidal neurons or interneurons. The authors should discuss these limitations, and possible impact on their assessment of selectivity against other VGSCs in their native cellular backgrounds.

There are pros and cons to any method of determining selectivity and we acknowledge that none of them are ideal for all purposes. We chose to focus on what we refer to as “molecular selectivity,” the fundamental ability for a compound to bind to the channel and stabilize the high affinity conformation. We accomplish this by choosing voltages that promote the same fraction of channels to be in the high affinity (inactivated) state. This contrasts with “functional selectivity” that may be largely driven by the distinct state-dependence of different isoforms. Our approach avoids assumptions about what the physiologically relevant voltage is since that voltage can vary depending on the tissue or cell type. For any given isoform there may be multiple physiologically relevant voltages.

Consistent with this philosophy, we bias all the channels to be in their highest affinity state (inactivated) and then use this maximal potency to compare selectivity. At more hyperpolarized, voltages, potency for all isoforms will tend to be somewhat less. We are adding more explanation of our rationale to the text, and we are adding supplemental data giving more insight into the impact of voltage on potency.

Figure 1—figure supplement 2 shows the potency of NBI-921352 after holding at a membrane potential nearer the physiologic range (-62mV). Potency at this voltage (IC_50_ = 53 nM) was similar to that at fully inactivated potentials evaluated in the primary potency assay described shown in Figure 1. For this reason, we anticipate that the selectivity ratios described in the manuscript will be similar to those in physiologic conditions.

A note to this effect has been added to the Results section. See line 126.

3) While the electrophysiological data clearly show that NBI-921352 shows voltage-dependence and likely state dependence, the study lacks an assay of use-dependence using repetitive higher frequency depolarizations. Something like 10 or 20 Hz pulsing might be informative.

We are in the process of preparing a manuscript on related compounds that will show a more detailed evaluation of the kinetics and state dependence of inhibition of Na_V_1.6. We have not yet explored this behavior as thoroughly with NBI-921352, and we feel that a more fulsome treatment of the biophysics may distract from the selectivity and pharmacology that we intend as the focus for the present work. We do know from our work in progress that block equilibrates over several seconds, and thus we do not expect a great deal of use-dependence at physiologically relevant firing rates.

4) In several places it is stated that NBI-921352 preferentially inhibits activated (inactivated or open) channels. There is no direct assay looking at inhibition of open but not-inactivated channels, so this conclusion is potentially misleading. The authors could try something like a WCW non-inactivating construct, but I think just rewording those places where it says inactivated or open to maybe "inactivating and possibly also open" would be reasonable.

Our data suggests preferential binding to inactivated states, but as the reviewer rightly points out, we cannot rule out (or in) binding to open states. Our intent with the “inactivated or open” wording was to account for this possibility. We are changing the wording to that recommended by the reviewer in hopes of clarifying this point. Our experience with constructs that impair fast inactivation (like WCW) have not been helpful for clarifying this issue because we find that these changes accelerate slow inactivation, and our compounds do not appear to clearly distinguish between slow and fast inactivated channels. Changes throughout document to replace “inactivated or open.”

5) Different voltage-clamp protocols were adopted among different sodium channel isoforms (lines 406-418) to measure IC50 concentrations of the drug, which requires clarification/justification. Since NBI-921352 is "a state-dependent inhibitor" (line 30) and "requires several seconds to equilibrate with activated channels" (line 401), the difference in holding potentials (-60 mV vs -45 mV), recovery period (20 ms vs 60 ms at -150 mV), and data acquisition rates (1 Hz vs 0.04 Hz) may alter the binding/interaction of NBI-921352 with tested channels, and therefore, introduce bias in the comparison of selectivity among VGSCs. The authors should explain their rationale for choosing these protocols and address the potential confounding effects in data analysis.

This question relates to the same underlying issues as Essential Question #2. I believe that the changes we made for #2 will also address this question.

6) The statement that a preference for persistent currents is, "in fact, a feature of all the compounds in the Nav inhibitor class" is not justified by either data presented in the study or by an appropriate reference. The "in fact" is particularly not justified since it really states a bias that would need to be bolstered by hard data. Not much in biology is proven as a fact. This bold statement may have some validity, but it is not really important to the major conclusions of the study.

We have altered our language to allow for the uncertainty pointed out by the Reviewer. See line 191.

7) It is stated that NBI-921352 is rapidly cleared in mice, but it is unclear if this is different in humans, which could limit the translational potential. It would help the reader if this was discussed.

Rodents, both rats and mice, rapidly eliminate NBI-921352, but the compound is much more stable in humans, where the half-life of elimination is approximately 8.5 hours. We have added this information to the Results section. See line 277.

8) Lines 248-249 states unequivocally that the efficacy and adverse events of Nav inhibitors is driven by action in the CNS. There is no citation for this and it comes across as another bias. It seems intuitive that the anti-seizure activity is dependent on CNS activity, but this is not demonstrated in the current study. Adverse events could involve sodium channels in muscle and/or the peripheral nervous system. Therefore this statement should be modified.

We agree and have modified the language to account for this uncertainty. See line 322.

9) Details on what behaviors were monitored and how they were monitored for figure 7 need to be included or else the bars and discussion on this aspect of the study should be removed.

Animals were observed by the scientist(s) responsible for dosing for the first 30 minutes after dosing for and again at the time of assay (2 hrs post dose) for signs of abnormal behavior. Abnormal behavioral signs were noted for all compounds – tremor, ataxia and hypoactivity – when plasma concentrations were sufficiently high. The lowest concentration at which such behavioral signs were noted in any of the animals tested is illustrated by the dotted line in figure 7. We have added more detail to the methods (see line 790) to clarify this point.

10) Nav1.6 is highly expressed in Purkinje neurons and motor neurons, and plays important roles in motor system. Did the authors observe any motor impairment in the behavior studies? It would have been informative to examine the effect of NBI-921352 on AP firing and resurgent currents in Purkinje neurons. As Nav1.6 is the major isoform found at nodes of Ranvier in myelinated fibers, including motor neurons axons, it is critical to know if NBI-921352 alters motor neuron and/or motor activity.

As noted in the response to Essential Revision #9, we did see signs of motor impairment at sufficiently high plasma concentrations of NBI-921352. While we cannot assure that these motor effects are due to inhibition of Na_V_1.6, we agree that it is consistent with the known role of Na_V_1.6 in the Purkinje neurons and in the nodes of Ranvier of motor neurons. Mice with sufficient knock down of Na_V_1.6 by genetic interventions are also known to exhibit motor dysfunction. Our data suggest that seizure protection occurs at lower concentrations/occupancy than those that induce ataxia. We believe that sparing other central Na_V_’s (Na_V_1.1 and Na_V_1.2) improves the profile of these inhibitors, but we do expect to see ontarget (Na_V_1.6 mediated) toxicity when receptor occupancy becomes too high. See additional discussion beginning at line 408.

11) Three seizure models were used to evaluate the effect of NBI-921352 on electrically induced seizure. The data shows that NBI-921352 prevented the GTC with hindlimb extension in all three assays, demonstrating its potential to become an antiepileptic drug. In an earlier work from the same group (J Med Chem 2019; 62: 9618-9641), a modified Racine scale (0-5) was used to score the severity of different seizure activities, while only GTC with hindlimb extension (score 5 in Racine scale) is considered as seizure in this study. Is there any reason to choose this evaluation criteria instead of Racine scale used in previous work?

We believe that both the Racine endpoint shown in Focken et al., 2019, and The GTC with hindlimb extension endpoint shown here are both valid. Our experience with this assay has been that the seizure phenotype is rather binary. That is, individual animals show very little seizure behavior prior to the GTC with hindlimb extension. As a result, we have found, based on studies with NBI-921352 and other compounds not shown here, that there is little difference in the efficacy measured by the method shown here versus the Racine evaluation used in the previous work. We chose to use the GTC with Hindlimb extension endpoint in this presentation to more easily compare and discuss the results in SCN8A mice to those presented for wild-type mice and rats in the DC-MES assays.

We have added the Racine score evaluation for these experiments in Figure 5—figure supplements 1 and 2, Figure 6—figure supplements 1, 2, and 3 to enable comparison with the SCN8A mouse fraction seizing results shown in the primary figures.

12) The authors tested seizure behaviors after repeated dosing of the drug, but claimed that there was no statistically significant difference between single-dose group vs repeated-dose group (lines 238-246). I was unable to find the description of statistical analysis method for behavioral studies in the manuscript.

We are including supplementary figures (Figure 5—figure supplements 1 and 2, Figure 6—figure supplements 1, 2, and 3) to better illustrate the statistical evaluation of the dose response data sets for all the experiments, and we have amended the text to indicate this. See Methods line 798, and the legends for Figure 5, Figure 5—figure supplements 1 and 2, Figure 6, and Figure 6—figure supplements 1, 2, and 3.

13) There are several problems in the brain slice recording experiments. First, paired student t-test was used to compare the AP firing before and after drug treatment, which is inappropriate. Repeated measures two-way ANOVA should be used here, or use area under curve rather than number of APs for statistical analysis. Second, despite the dominant effect of Nav1.1 in inhibitory interneurons, Nav1.6 is also expressed in interneurons, and to confirm selectivity in the mouse model, it is better to examine the effect of NBI-921352 on AP firing in Nav1.6-knockout neurons. Third, the experiments lack a vehicle control data set. Fourth, the number of cells for each group is low (3-4 cells), more recordings should be performed.

We have changed the statistical analysis to an area under the curve analysis as suggested by the reviewer. See text line 234, Figure 4 legend and transparency Excel data file. Na_V_1.6 knockout experiments would be of interest, but we do not have access to those neurons. We agree that cells would be desirable. Unfortunately, the pandemic disrupted these slice experiments. Subsequently, NBI-921352 advanced in its clinical development. This creates additional challenges and costs to adhere to regulatory reporting requirements and has prevented us from extending this dataset.

14) Please add a section of statistical analysis in Materials and methods, and list the statistical analysis method used in each experiment.

We’ve added statistical methods to the methods and to the figure legends where appropriate. See methods line 798, figure legends, and supplemental figure legends.

[Editors' note: further revisions were suggested prior to acceptance, as described below.]

The manuscript has been improved but there are some remaining issues that need to be addressed, as outlined below:

1. The reviewer 1 pointed out the lack of NBI-921352 data on Nav1.8 and Nav1.9, and the authors performed limited study on hNav1.8 co-expressed with β3 in CHO cells.

I am not clear why the authors selected CHO cells as the expression system, as it is known that expression of Nav1.8 is poor in CHO and HEK293 cells, even in the presence of β subunits (Nature 2002, 417: 653-656). The ND7/23 is a more commonly-used system for Nav1.8 expression (for example, Toxins 2021, 13:501). I have obtained decent Nav1.8 currents (over 10 nA, with properties similar to those measured in DRG neurons) in ND7/23 cells, but barely saw Nav1.8 currents in HEK 293 cells co-transfected with hNav1.8 and β3. It is unclear how good the Nav1.8 expression was in CHO cells in authors' hands, the authors should include raw traces of Nav1.8 in Figure 1-Supplement 3 to demonstrate the reliable Nav1.8 expression in this assay. If the expression of Nav1.8 is not good, I would recommend removing the Nav1.8 data, though the authors still need to explain the lack of data on those two isoforms and discuss possible effect of NBI-921352.

Typos: there are quite some typos in figure legend of Figure 1-Supplement 3. For example, line 1045, "% Block (Tonic) = (1 ITPI,NBI921352 / ITPl,Control) X 100%", should "ITPl" be "ITP1"? And the calculation should be "(1-ITP1,NBI921352/ITP1,Control) x 100%"? Same typo is shown in line 1046.

In line 1049, "% Block (inactivation state) -", should "-" be "="?

The primary reason we did not pursue experiments with Na_V_1.8 or Na_V_1.9 for this work is that those channels are not directly relevant to our primary goal of understanding sodium channel inhibition in epilepsy. Both Na_V_1.8 and Na_V_1.9 are largely restricted to peripheral neurons and are not expected to impact the efficacy of sodium channel inhibitors as antiseizure drugs. We also do not expect inhibition of Nav1.8 or Nav1.9, if it were to occur, to create safety liabilities.

For these reasons we chose not to invest in developing assays for these channels for this work. We have endeavored to make this clear in the manuscript.

The Na_V_1.8 data provided was performed at a commercial laboratory (Charles River) and the assay is not well matched to our own internal assays. This mismatch was the reason that we initially excluded the data from the manuscript. We will remove the Na_V_1.8 data since it does not impact our conclusions.

Our apologies for the typographical errors in the legend, we have removed it since the Na_V_1.8 data in Figure 1—figure supplement 3 will no longer be included in the manuscript.

We will address question 3 before question 2 since the response to question 3 informs our response to question 2.

3. Regarding to the state of channels that NBI-921352 preferentially inhibits, the abstract wrote "NBI-921352 is a state-dependent inhibitor, preferentially inhibiting inactivated channels", while the figure legend 3 shows "NBI-921352 is a state-dependent inhibitor of NaV1.6 and preferentially inhibited activated (open or inactivated) channels". In main text (page 20, line 424-426), "NBI-921352 is also highly state dependent, with a >750-fold preference for activated channels vs. rested, closed-state channels (sometimes referred to as peak current)". Which state does NBI-921352 preferentially act?

We believe that NBI-921352 preferentially binds to inactivated channels. This is based on the failure of the compound to potently block rested (closed) channels. Also, the presumed binding site (the UP, activated, conformation of the Domain IV voltage sensor, VSD4) only becomes available once VSD4 has reacted to the membrane field to trigger channel inactivation (see Ahuja et al., 2015, McCormack et al., 2013). We have not done detailed studies to exclude the possibility that open channels can be bound, though it seems unlikely to be a major driver of inhibition based on the limited time that the channels are in open, relative to inactivated, states. While the assays are designed with a bias toward inactivated states, we have not attempted to differentiate between fast and slow inactivated state inhibition. Our main objective for this manuscript is to define the importance of selective inhibition of Na_V_1.6 for treating epilepsy. We expect further clarification of the interaction of drug binding and channel gating will be a topic for future work.

We have corrected the language in the manuscript and in the legend for Figure 3 to make clear that NBI-921352 targets inactivated states but that resolution between open, fast inactivated, and slow inactivated states was not attempted.

2. I have concerns on different holding potentials being used in determining IC50 of NBI-921352 for different isoforms. The authors responded that "We chose to focus on what we refer to as "molecular selectivity," the fundamental ability for a compound to bind to the channel and stabilize the high affinity conformation. We accomplish this by choosing voltages that promote the same fraction of channels to be in the high affinity (inactivated) state." I wonder how the authors determined that "-45 mV" is the potential for NBI-921352 to bind and reach the high affinity conformation of Nav1.1, 1.2, 1.3, 1.4, and 1.6, and "-60 mV" for Nav1.7 and Nav1.5, there is no justification of this in the manuscript. In addition, when a medicine is applied to a patient, the adverse effect is determined by its affinity and effect on all targeted channels/molecules physiological or pathological conditions, therefore, the relative selectivity of NBI-921352 determined using the same protocol/condition would be more meaningful for clinical practice.

The new data that NBI-921352 showed similar potency in Nav1.6 channel at -62 mV when compared to that at -45 mV is important, this data suggests that the IC50 of NBI-921352 is not significantly changed by the holding potential (or membrane potential) between -62 mV and -45 mV. Although I don't agree with the authors' explanation, I will not ask for extra experiments. However, unless the authors provide convincing evidence that the two holding potentials (-45 mV and -60 mV) used in this study do allow different isoforms to be in their "highest affinity state (inactivated)", the explanation in Page 6 (line 124 to line 138) needs to be modified/removed.

We were fortunate that the voltage dependence of gating for the channels most relevant to our seizure studies (the CNS channels) aligned well enough to enable a common voltage protocol for all 4 CNS Na_V_ channel isoforms. The conclusions on the advantage of selective inhibition of NaV1.6 for treating epilepsy are most dependent upon this selectivity profile and for these isoforms molecular selectivity and functional selectivity coincide.

As indicated in the response to question 3, NBI-921352 prefers inactivated channels. To create a robust assay suitable for screening compounds (about 2000) over the course of several years, we sought to create assays that were highly robust and reproducible over time. We have found that this is sometimes not possible with a single voltage protocol for every channel of interest.

To achieve robust and sensitive assays, we hold the membrane potential positive enough to ensure that most channels are inactivated. We then briefly step to a hyperpolarized potential (150 mV) that allows fast inactivated channels to recover but doesn’t allow for recovery from slow inactivation. If drug binding stabilizes the inactivated state sufficiently then drug bound channels also do not recover – leading to current inhibition. Holding at potentials that are too depolarized leads to an accumulation of slow inactivated channels and causes rundown. This reduces the size of the current signal and increases the challenge of separating gating drift from compound inhibition. These issues can compromise the fidelity and reproducibility of the inhibition assay. We empirically determined that the voltages used here result in full inactivation and are stable enough to enable robust measures over the duration of the assay.

For most isoforms (Na_V_1.1, Na_V_1.2, Na_V_1.3, Na_V_1.4, and Na_V_1.6) -45 mV was found to have complete inactivation and a stable recovered current amplitude. The isoforms that inactivate at the most negative potentials (Na_V_1.5 and Na_V_1.7) needed a more negative holding potential (60 mV) to preserve robust assay performance while fully inactivating the channels.

While unlikely to impact efficacy, Na_V_1.5 inhibition is important from a safety perspective as inhibition of Na_V_1.5 would introduce risk of cardiac events. Cardiac cells generally have more negative resting membrane potentials (around -90 mV) than neurons so inhibition in cardiac tissue will likely be less potent than inhibition in our assay.

We have removed the section (lines 124-138) indicated by the reviewer and instead added more text on protocols to the beginning of the section, “NBI-921352 is a state-dependent inhibitor” to improve the clarity of our methods and rationale for the readers.

Typo: line 137, "is this assay" should be "in this assay".

This typo was removed.

4. The figure legend 5 is confusing (page 63, line 1159-1169), please rephrase the sentences.

We have expanded the legend for Figure 5 to clarify.
